# Bacterial profiling of *Haemonchus contortus* gut microbiome infecting Dohne Merino sheep in South Africa

**DOI:** 10.1038/s41598-021-85282-7

**Published:** 2021-03-15

**Authors:** T. Mafuna, P. Soma, A. M. Tsotetsi-Khambule, C. A. Hefer, F. C. Muchadeyi, O. M. M. Thekisoe, R. E. Pierneef

**Affiliations:** 1 Biotechnology Platform,, Agricultural Research Council, Private Bag X05, Onderstepoort, 0110 South Africa; 2 Animal Production, Agricultural Research Council, Private Bag X2, Irene, 0062 South Africa; 3 Lincoln Research Center, AgResearch Ltd, 1365 Springs Road, Lincoln, 7674 New Zealand; 4 Center for Bioinformatics and Computational Biology, Department of Biochemistry, Genetics and Molecular Biology, University of Pretoria, Lynwood Road, Pretoria, 0001 South Africa; 5 Department of Life and Consumer Sciences, University of South Africa, Florida Campus, Private Bag X6, Florida, 1710 South Africa; 6 Unit for Environmental Sciences and Management, North-West University, Private Bag X6001, Potchefstroom, 2520 South Africa

**Keywords:** Metagenomics, Metagenomics

## Abstract

A metagenomic approach was used to study the gut microbiome of *Haemonchus contortus* field strains and that of its predilection site, the abomasum of Dohne Merino sheep. The abomasum contents and *H. contortus* were collected from 10 naturally infected Dohne Merino sheep. The *H. contortus* specimens were classified and sexually differentiated using morphometric characters and was further confirmed through molecular identification. We investigated differences and similarities between the bacterial composition of the adult male and female *H. contortus* gut microbiomes, which were both dominated by bacteria from the *Escherichia, Shigella, Vibrio* and *Halomona*s genera. Major abundance variations were identified between the shared adult male and female *H. contortus* microbiomes. The results also revealed that *Succiniclasticum, Rikenellaceae* RC9 gut group and *Candidatus Saccharimonas* were the predominant genera in the Dohne Merino abomasum. This study provides insight into the highly diverse bacterial composition of the *H. contortus* gut microbiome and the Dohne Merino abomasum which needs to be studied further to explore the complex interactions of different gastrointestinal nematode microbiomes with the host.

## Introduction

Microbiomes are a complex community of microorganisms that vary in their occupied niches, species composition and their influence on various host environments^1^. These microorganisms live in a close symbiotic relationship with eukaryotic host organisms including nematodes, insects, plants, animals and humans^2^. The symbiotic relationship between microbes and their hosts range from commensalism, mutualism, parasitism and fatal pathogenic infections^3,4^. The most extensively researched symbionts of nematodes are those of parasitic plant nematodes and filarial worms, mostly due to their agricultural, biological and medical significance^4,5^. The most researched symbionts of small ruminants are those of domestic sheep and goats as well as other nematode-bacterial and small ruminant-bacterial symbioses of agricultural interest^6,7^.

The microbiome occurring within an animal’s gastrointestinal tract (GIT) plays an important role in digestion, nutrition, metabolism and immunity of their host^2^. Gastrointestinal nematodes (GIN) have a distinct microbiome from their host, and the study of the symbiotic relationship between this microbiome and the GIN host has grown rapidly in recent years, with previous studies reporting on the microbiome associated with *Trichuris muris*^8^, *Ascaris suum*^9^ and *H. contortus*^4,10^. Most of these studies conclusively demonstrate that GIN, in particular the *H. contortus* harbour microbiome associated with the nematode’s egg-, larval- and adult worm stages performing various functions during their life cycle stages^4,10^.

The GIN parasite-microbiome symbioses have a measurable impact on the utilization of nutritional resources, maintenance of homeostasis in the digestive tract, and host health^10,11^. Previous studies on GIN parasite-microbiome interactions have predominantly focused on establishing the microbiome of the host GIT, and the characterization of the microbiome functional roles in metabolism, nutrition and immunity as well as the impact of GIN parasites on host microbiome^12^. Currently, there is a lack of scientific information on the microbiome harbored by GIN parasites of livestock and their relationship to the host microbiome.

The control of GIN parasites is primarily based on the administration of anthelmintic drugs. The extensive use of anthelmintic drugs leads to the development of drug resistance in GIN parasites, especially the barber’s pole worm *H. contortus*, which is a highly pathogenic and probably the most economically important nematode infecting small ruminants^4,10^. Hence, alternative control measures are needed to effectively control GIN parasites. A novel approach based on biocontrol using symbiotic microbial communities has been suggested to limit the use of chemical based treatments^13^. As such, it is important to understand the dynamics of GIN infections and their respective microbiome in comparison to that of the host which will contribute valuable information towards exploiting the microbiome in an attempt to control GIN in small ruminants^4,10,14^. The current study focused on the characterization of the adult male and female *H. contortus* microbiome and that of their predilection site, the abomasum of Dohne Merino sheep.

## Results

### Morphometrics and molecular analysis of *H. contortus* isolated from Dohne Merino sheep

A total of 266 GIN, 169 females (63.5%) and 97 males (36.5%) were recovered from 7 out of 10 (70%) abomasum of the Dohne Merino sheep sampled (Table S1 and Fig. S1). A total of 7 pooled adult male *H. contortus* and 7 pooled female *H. contortus* samples identified by their morphometric characteristics were confirmed by PCR assay to be *H. contortus* with an amplification band present at 260 bp (Fig. S3). Care was taken to restrict microbiome contamination (i.e. intact bacteria and their DNA products) originated from the abomasal microbiome (Fig. S2).

### Alpha diversity indices of the adult male and female *H. contortus* and abomasum microbiome

**Figure 1:**
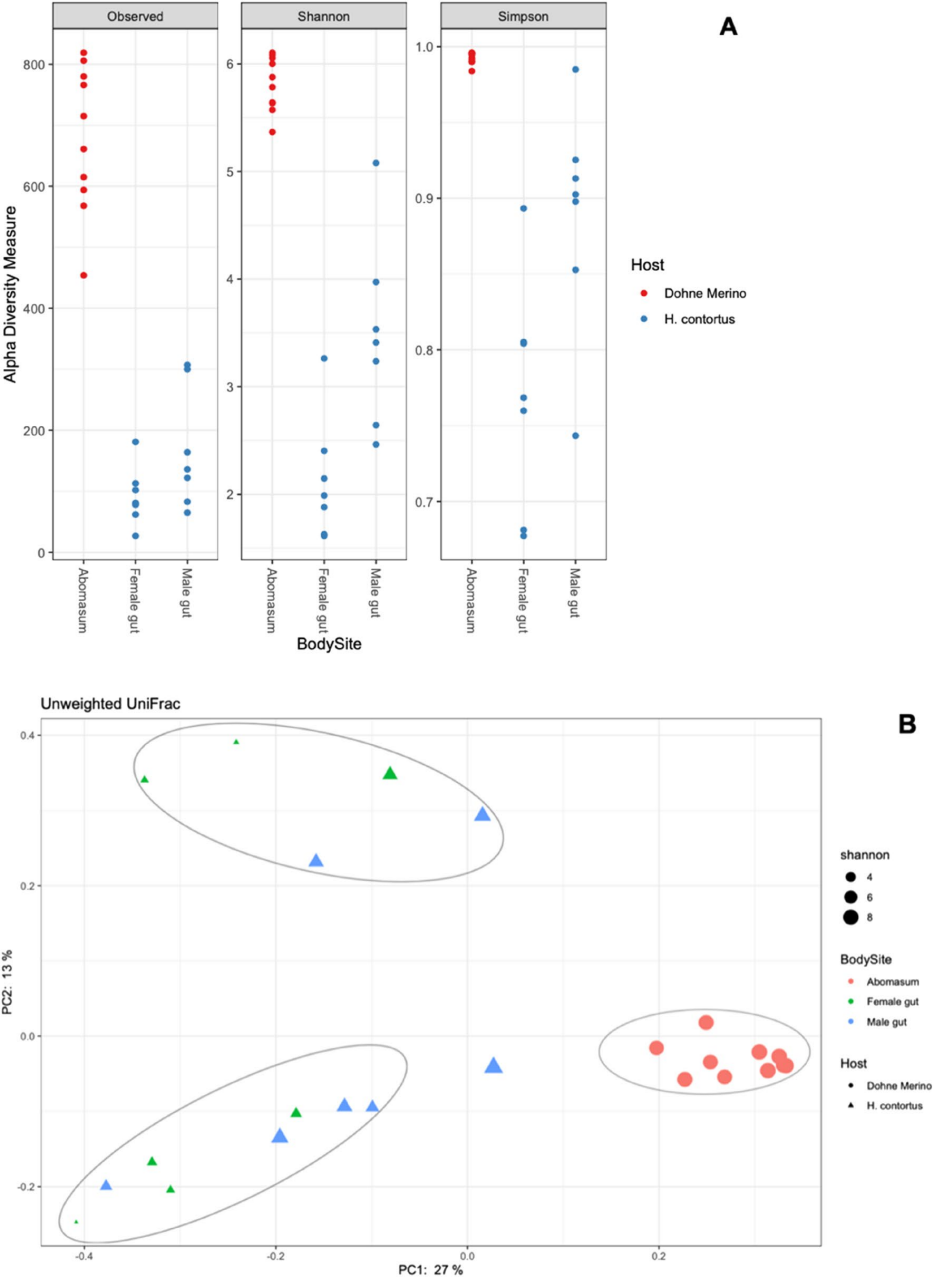
Principal coordinate analysis (PCoA) based on unweighted UniFrac distances (**B**). Alpha diversity (**A**) matrices, observed OTUs, Shannon, Simpson of the abomasum content, adult male and female *H. contortus* microbiome at 97% identity. Data represent average values from pooled populations.

As shown in Table 1, the adult male *H. contortus* had a higher Operational Taxonomic Unit (OTU) richness than the female (*p*-value < 0.05). However, when compared to the abomasum, the abomasum microbiome had the highest richness (*p*-value < 0.05). There was a clear difference between the adult male *H. contortus* gut microbiome in comparison to the female gut microbiome across all alpha diversity indices except for Faith’s Phylogenetic Diversity (PD) which did not show any significant difference (*p*-value > 0.05) (Table 1 and Fig. S4). Similarly, in Fig. 1A, the abomasum content was compared to the adult male *H. contortus* and the female microbiome, and displayed a significant difference in Observed OTUs, Shannon and Simpson indices (*p*-value < 0.05).

**Table 1:** Overview of alpha diversity metrics for the adult male, adult female *H. contortus* and abomasum content microbiome.

Diversity matrices	Abomasum microbiome*	*H. contortus* male Microbiome*	*H. contortus* female microbiome*	*Abomasum* vs *H. contortus* male		*Abomasum* vs *H. contortus* female		*H. contortus* male vs *H. contortus* female	
	Average; STD	Average; STD	Average; STD	H-value	*p *value	H-value	*p* value	H-value	*p *value
Observed OTUs	1144.4 ± 268.091	339.571 ± 155.680	159.714 ± 110.045	12	0.000636	12	0.000636	4.4	0.035006
Shannon-index	8.906 ± 0.421	5.815 ± 1.267	3.274 ± 1.049	12	0.000636	12	0.000636	8.3	0.004041
Simpson’s index	0.994 ± 0.003	0.913 ± 0.062	0.777 ± 0.080	12	0.000906	12	0.000636	6.9	0.008809
Faith_pd	70.50 ± 15.930	38.639 ± 15.790	33.260 ± 22.530	8	0.004653	9	0.003415	0.5	0.482203
Genus evenness	0.884 ± 0.0196	0.698 ± 0.1147	0.464 ± 0.0854	12	0.000636	12	0.000636	8.3	0.00404

*P* values were calculated using Kruskal–Wallis test. The level of significance was determined at *P* < 0.05. * Data represent average and standard deviation (STD) values from pooled populations.

### Beta diversity indices of the adult male and female *H. contortus* and abomasum microbiome

The Principal Coordinates Analyses (PCoA) results indicated that adult male and female *H. contortus* microbiome clustered together. The abomasum microbiome clustered on its own and was separated from the adult male and female *H. contortus* microbiome cluster (Fig. 1B). Furthermore, the unweighted (Fig. 1B) and weighted UniFrac (Fig. S5) distance analysis indicated that the microbiome composition of the abomasum was significantly different from that of the adult male and female *H. contortus* (*p*-value < 0.05) (Table S3). The microbiome composition was also significantly different between the abomasum content and the adult female *H. contortus* (*p*-value < 0.05). The abomasum and adult male *H. contortus* microbiome was also found to be significantly different (*p*-value < 0.05) (Table S3).

### Microbiome composition at the phylum level

**Figure 2:**
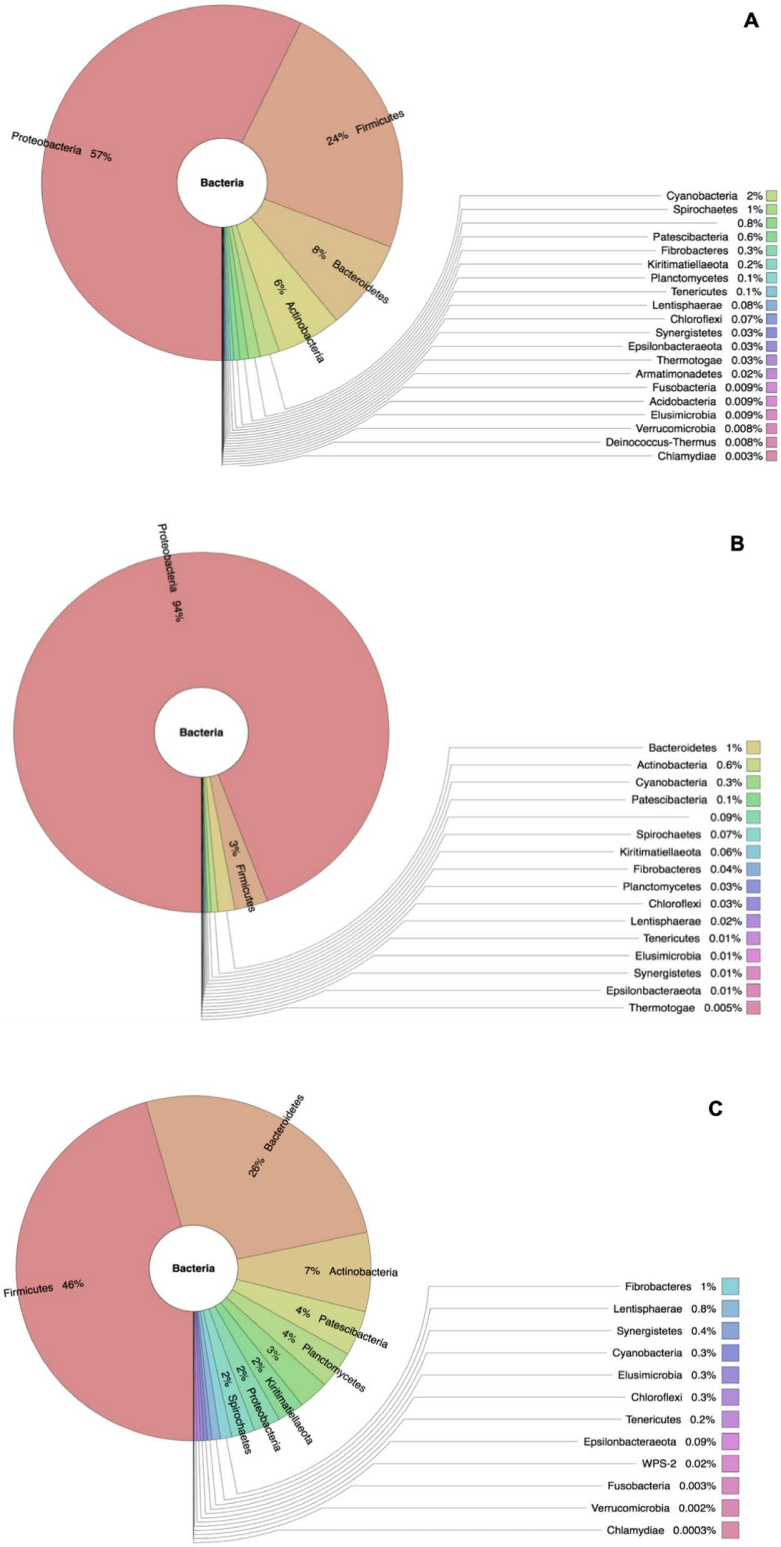
Krona plot for taxonomic abundance of the adult male (**A**), female (**B**) *H. contortus* and Abomasum (**C**) microbiome at the phylum level. Data represent average values from pooled populations.

A total of 23 different phyla were identified in the adult male *H. contortus* microbiome which were predominantly dominated by bacterial phyla: *Proteobacteria* (57%), *Firmicutes* (24%), *Bacteroidetes* (8%) and *Actinobacteria* (6%) (Fig. 2A). In the adult female *H. contortus* microbiome, a total of 17 different phyla were identified with the dominant phyla being *Proteobacteria* (94%) followed by *Firmicutes* (3%), *Bacteroidetes* (1%), *Actinobacteria* (0.6%) and *Cyanobacteria* (0.3%) (Fig. 2B). Analysis of the abomasum identified a total of 20 different phyla. The five most abundant bacterial phyla in the abomasum were *Firmicutes* (45%) followed by *Bacteroidetes* (26%), *Actinobacteria* (7%), *Patescibacteria* (4%) and *Planctomycetes* (3%) (Fig. 2C).

### Microbiome composition at the genus level

**Figure 3:**
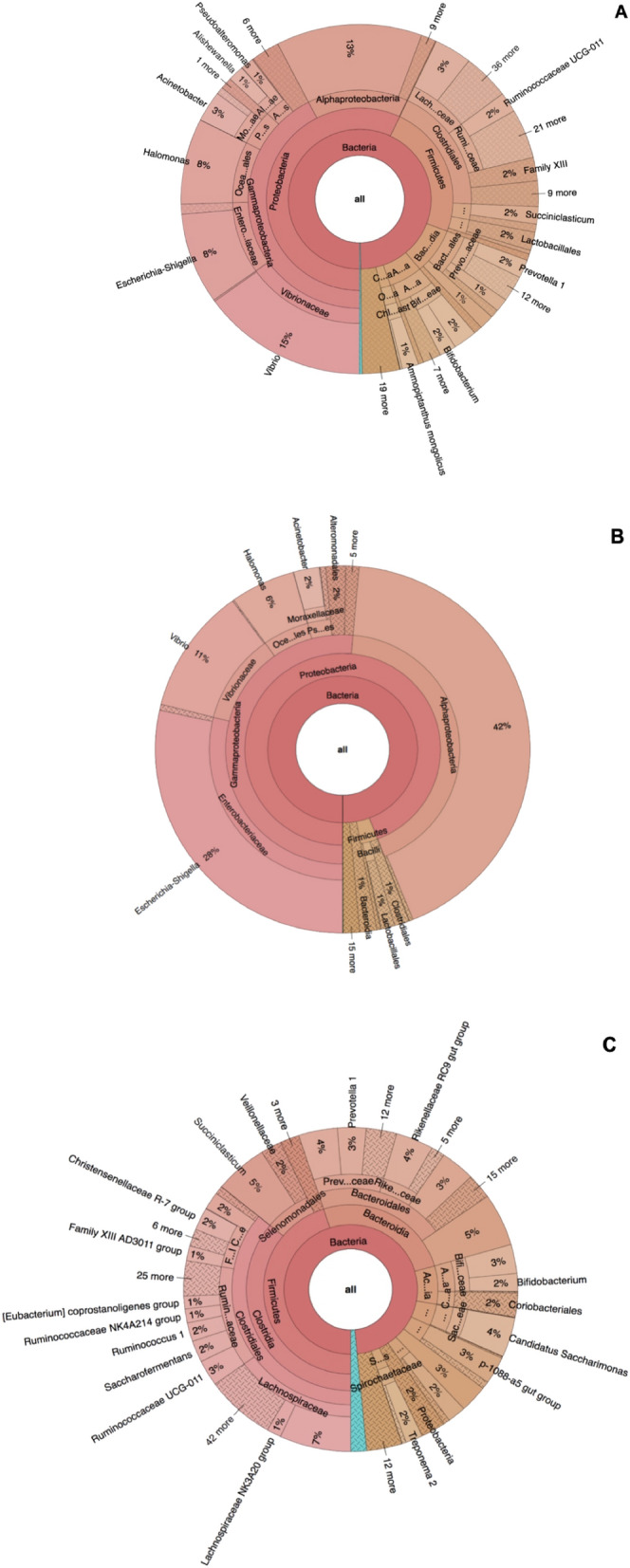
Krona plot for taxonomic abundance of the adult male (**A**), female (**B**) *H. contortus* and Abomasum (**C**) microbiome at the genus level. Data represent average values from pooled populations.

The adult male *H. contortus* microbiome was dominated by *Vibrio* (15%) followed by *Escherichia-Shigella* (8%), *Halomonas* (8%) and *Acinetobacter* (3%) (Fig. 3A). The predominant genera in the adult female *H. contortus* microbiome was *Escherichia-Shigella* (28%), *Vibrio* (11%), *Halomonas* (6%), *Acinetobacter* (2%) and *Alteromonadales* (2%) (Fig. 3B). The abomasum was dominated by *Succiniclasticum* (5%), *Rikenellaceae* RC9 gut group (4%), *Candidatus Saccharimonas* (4%), *Ruminococcaceae* UCG-011 3%, p-1088-a5 gut group (3%) and *Prevotella* 1 (3%) (Fig. 3C).

### Unique and shared microbiome composition

**Figure 4:**
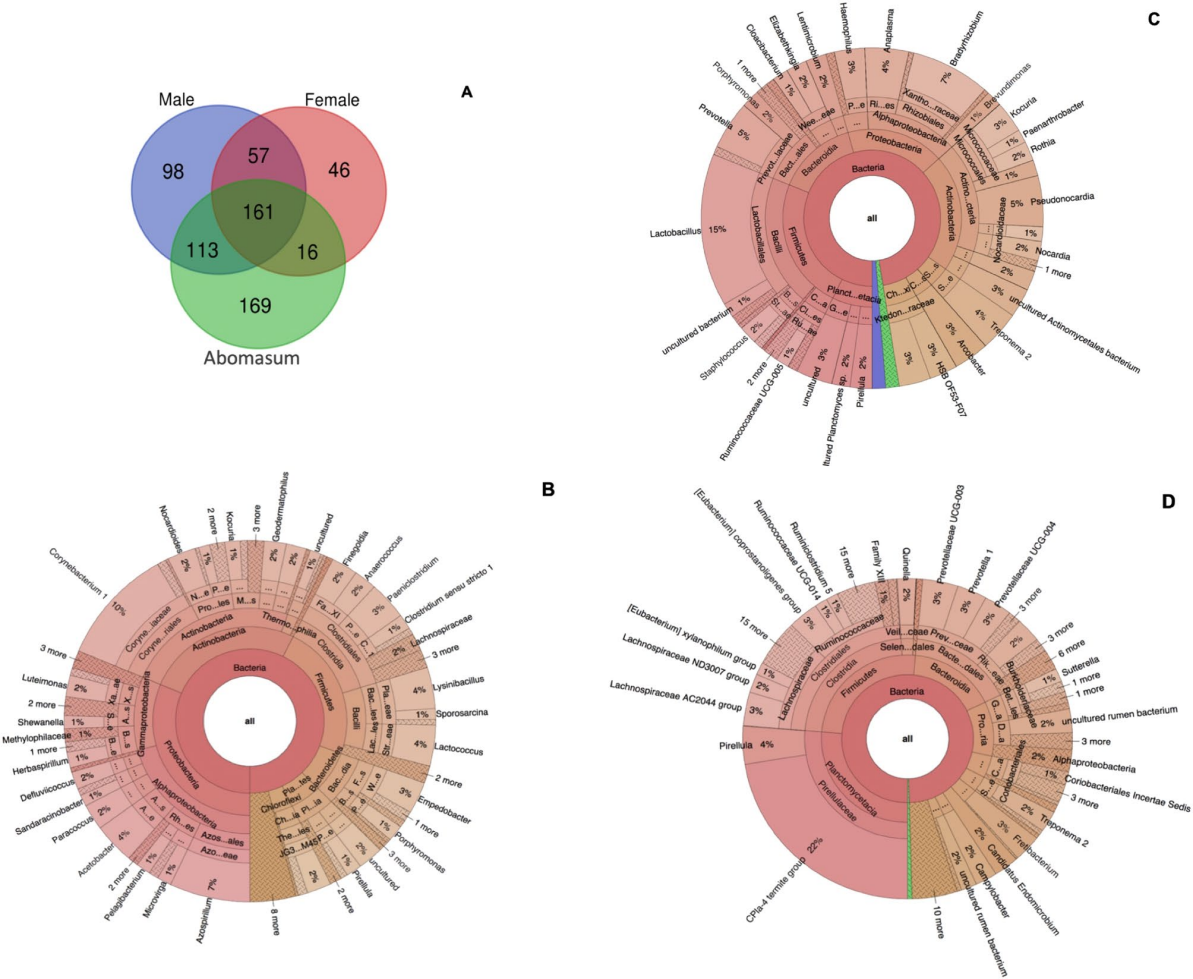
Venn diagram (**A**) and krona plot depicting the composition and number of unique and shared OTUs associated with adult male (**B**), female (**C**) *H. contortus* and abomasum content (**D**) microbiome. Data represent average values from pooled populations.

In total, 660 unique bacterial OTUs across the three hosts were identified (Table S4). The abomasum content had higher bacterial OTU abundance followed by the adult female *H. contortus* whilst the male had the lowest bacterial OTU abundance (Fig. S6). The results showed a total of 57 bacterial OTUs shared between the adult male and female *H. contortus*. The adult male *H. contortus* and abomasum shared 113 OTUs with each host having 98 and 169 unique OTUs present, respectively. A total of 16 bacterial OTUs were shared between adult female *H. contortus* and the abomasum (Fig. 4A).

### Unique genera in the microbiome

The unique genera in the adult male *H. contortus* were found to be *Corynebacterium* 1 (10%), *Azospirilum* (7%), *Lysinibacillus* (4%), *Lactococcus* (4%), *Acetobacter* (4%), *Empedobacter* (3%) and *Paeniclostridium* (3%) (Fig. 4B). The adult female *H. contortus* contained the following unique genera: *Lactobacillus* (15%), *Bradyrhizobium* (7%), *Pseudonocardia* (5%), *Prevotella* (5%), *Anaplasma* (4%) and *Treponema2* (4%) (Fig. 4C). Unique genera in the abomasum were *CPla-4 termite group* (22%), *Pirellula* (4%)*, Lachnospiraceae* AC2044 group (3%), *Prevotellaceae* UGG003 (3%), *Prevotellaceae* UGG004 (3%), *Prevotella* (3%) and *Coprostanoligenes* group (3%) (Fig. 4D).

## Discussion

The barber’s pole worm (*H. contortus*) is a common parasitic nematode infecting small ruminants and one of the most pathogenic GIN of sheep^15–17^. Identification of common stomach worms, including *H. contortus,* in small ruminants has been previously based on recognized morphological characteristics that make it possible to differentiate between *Haemonchus* species, particularly, *H. contortus* and *H. placei*. The spicule lengths and vulvar flap provide the quickest and easiest characteristics to be used for separating populations of *H. contortus* from *H. placei*.^18–20^. In the present study, the recovered *Haemonchus* spp. specimens were identified as adult male and female *H. contortus* based on their morphological characteristics as suggested by Lichtenfels et al.^21^ and Gibbons^18^. The adult female *H. contortus* were recognised mainly by their reddish colour and vulvar flap features. The adult male *H. contortus* were identified based on the low variability of spicule morphology within species. The morphological classification of the specimens was confirmed by PCR assay using species specific *H. contortus* primers ^22^.

Following recovery of the parasites from the abomasum, a rigorous treatment consisting of antibiotic solution (1 mg/ml ampicillin/1 mg/ml gentamicin) and 4% sodium hypochlorite was used to ensure removal of all microbiome contamination on the parasite surface. The PCR assay results with 16S rRNA gene primers further served as an indicator that there was no contamination on the parasite originating from the abomasal microbiome. These results corroborated the previously published results by White^23^ and El-Ashram and Suo^10^ who reported that sterilisation with this method is sufficient to remove abomasal microbiome contaminants on the surface of the GIN.

The adult male and female *H. contortus* microbiome were characterised in the absence of external microbiome contaminants on the surface of the parasite. The results indicated that the adult female *H. contortus* microbiome was dominated by bacterial phyla: *Proteobacteria* (94%) followed by *Firmicutes* (3%), *Bacteroidetes* (1%), *Actinobacteria* (0.6%) and *Cyanobacteria* (0.3%). The adult male *H. contortus* microbiome was dominated by bacterial phyla: *Proteobacteria* (57%) followed by *Firmicutes* (24%), *Bacteroidetes* (8%), *Actinobacteria* (6%) and *Cyanobacteria* (2%). These results are similar to those observed in earlier studies conducted on *Trichuris muris* by White^23^, who reported that the adult *T. muris* microbiome was dominated by bacterial phyla: *Bacteroidetes* followed by *Firmicutes* and *Proteobacteria*. El-Ashram and Suo^10^ and Sinnathamby et al.^4^ reported that the *H. contortus* microbiome was dominated by the bacterial phyla: *Proteobacteria* followed by *Firmicutes, Tenericutes* and *Actinobacteria*, and *Firmicutes* followed by *Proteobacteria* and *Bacteroidetes* respectively. Relatedly, the microbiome composition at phylum level observed in the current study on *H. contortus* were similar to those observed in previous studies of *T. muris, Ascaris suum* and *H. contortus* from United Kingdom, United States of America and China respectively. However, there were variations in abundance of these phyla which are usually influenced by the type of host they inhibit and environmental conditions^10,23^. White^23^, El-Ashram and Suo^10^ and Sinnathamby et al.^4^ compared the differences and similarity in the microbiome of the different parasite life-cycle stages but not of the different genders of the adult stages and the host. This study therefore provided additional information by comparing the adult male and female *H. contortus* microbiome as well.

On a genus level, the adult female *H. contortus* microbiome was dominated by bacterial genera: *Escherichia-Shigella* (28%) followed by *Vibrio* (11%), *Halomonas* (6%), *Acinetobacter* (2%) and *Alteromonadales* (2%). The dominant genera in the adult male *H. contortus* microbiome were *Vibrio* (15%) followed by *Escherichia-Shigella* (8%), *Halomonas* (8%) and *Acinetobacter* (3%). The predominant bacterial genera in the adult male *H. contortus* were similar to female but with variation in the genera abundance. The adult female *H. contortus* showed higher genera abundance than male *H. contortus*, although the adult male *H. contortus* had more diversity than female in terms of the overall microbiome. Among the aforementioned genera, *Escherichia- Shigella, Vibrio, Halomonas* and *Acinetobacter* were predominantly shared by both adult male and female *H. contortus*. These results are different from those reported by El-Ashram and Suo^10^ who indicated that the predominant bacterial genera of the *H. contortus* were *Escherichia-Shigella* (87.5%), *Pseudomonas* (5.91%) and *Ochrobactrum* (5.06%), which we believe is caused by the type of host they infect and environmental conditions of their locations.

The microbiome of the adult male and female *H. contortus* showed similarity and variations in terms of abundances and diversity in the present study. These results indicated that *H. contortus* harbours a distinct microbiome compared to their host. There was significant difference between adult male and female *H. contortus* microbiome diversity and abundances. The *H. contortus* samples in this study were recovered from the same sheep breed, fed under the same diet and under similar environmental conditions. The variations in the microbiome of adult male *H. contortus* and female may be caused by hormonal changes, immune response or the reproductive needs, including egg development in females, which does not occur in male GINs. The presence of the microbiome in *H. contortus* suggests a potential nutritional importance of the microbiome to the worms, either providing metabolites that the worm cannot make itself, or aiding the breakdown of ingested blood meal.

The Dohne Merino abomasum microbiome naturally infected with *H*. *Contortus* field strains were characterised to gain insight into the impact that GIN parasites infection has on the host microbiome and how these interactions influence *H. contortus* microbiome. The abomasum microbiome was characterised and found to be dominated by the following bacterial phyla: *Firmicutes* (45%), *Bacteroidetes* (26%), *Actinobacteria* (7%), *Patescibacteria* (4%) and *Planctomycetes* (3%). Similar observations were reported in previous studies on small ruminants which indicated that the abomasum was dominated by *Bacteroidetes*, *Firmicutes, Actinobacteria* and *Proteobacteria*.^24,25^. With regard to the abundance of these predominant phyla, there were variations found across the studies. Interestingly, the phyla *Patescibacteria* and *Planctomycetes* were found in abundance in the abomasum content in the present study with none of these phyla reported in other previously published studies^23–26^. These variations between the present study and previously published studies could be due to known factors that affect microbiome composition such as parasite burden, breed type, diets and different environmental condition.

The predominant assigned bacterial genera observed in the abomasum content were *Succiniclasticum* (5%), *Rikenellaceae* RC9 gut group (4%), *Candidatus Saccharimonas* (4%), *Ruminococcaceae* UCG-011 3%, p-1088-a5 gut group (3%) and *Prevotella 1* (3%). These predominant genera are known to specialize in complex polysaccharides degradation, such as starch and cellulose, and contribute in the metabolism of dietary fiber in ruminants^27^. These results agree with observations reported by Zeng et al.^25^, Tanca et al.^28^ and Zhang et al.^27^ whereby predominant genera in the GIT depend on the parasite burden, diet and environmental condition. Hence, there were variations in the microbiome observed in the present study and previously published studies^25,27,28^.

The results further indicated that all abomasum content microbiome clustered together, suggesting that the sheep abomasum harbour similar microbiome. The adult male and female *H. contortus* microbiome clustered together, indicating that they harbour similar microbiome as well, which could be due to similar living environmental conditions and diet. El-Ashram and Suo^10^ reported similar results which shows the *H. contortus* eggs and adult stage microbiome cluster together because they share similar environmental conditions inside the host. When compared, the adult male *H. contortus* and female appeared to have lower bacterial diversity than the abomasum content. Hence, the *H. contortus* microbiome does not simply reflect their host abomasum content microbiome, although they spend majority of their life-cycle in the host abomasum. A previous study by Sinnathamby et al.^4^ reported that the different parasite life-cycle stages microbiomes are different from that of their predilection site. Moreover, El- Ashram and Suo^10^,Sinnathamby et al.^4^ also reported that during the *H. contortus* life cycle development stages, the egg stage, L1, and L2 and L3 interact with different microbiome from their respective environment, thus each stage might have distinct microbiome that they pick up from their environment and the host.

Furthermore, we found that the adult male *H. contortus* harbour a higher number of unique bacterial OTUs compared to the female and shared a higher number of bacterial OTUs with the abomasum content than the female. These results suggest that there are various selective measures at play within the *H. contortus* digestive tract, different to that of the host gastrointestinal tract. Therefore, some bacterial species are unable to persist and others thrive. The metabolic needs of the *H. contortus* might vary to that of its host and microbiome selection will depend on what GIN parasite require for its own fitness and survival within the host. These results correspond with Sinnathamby et al.^4^ observations whereby they reported that the *H. contortus* harbour distinct/unique microbiome from its host, which are important for their survival. Furthermore, the present study indicated that the abomasum content harbour higher OTUs abundance compared to *H. contortus*, which would be due to its higher metabolic needs. This observation is similar to previous studies on *T. muris* and *H. contortus* by White et al.^29^ and Sinnathamby et al.^4^ which reported that the host harbour more diverse microbiome than the parasite.

Previous studies by Lu et al.^30^,Gómez-Garzón et al.^31^ and Abdel-Salam et al.^32^ reported that bacterial strains, *Pseudoalteromonas marina, Vibrio attantica strain 5–16, Pseudomonas aeruginosa* and *beteli,* and *Lysinibacillus* can produce nematicidal volatile compounds with activities against the root-knot nematode *Meloidogyne incognita* and pine wood nematodes. Interestingly, similar bacterial genera were observed in the unique OTUs in the present study as well. As result, these bacteria can be manipulated in livestock production system due to their nematicidal activity to target pathogenic GIN in livestock such as *H. contortus* and potentially become biological control agents for GIN parasites while reducing the use of chemical based treatments^31,32^. The presence of these bacteria gives hope that it is possible to use symbiotic microbiome to control GIN parasites in livestock.

In conclusion, the adult male *H. contortus* appears to harbour more diverse microbiome as compared to the female. The abomasum has more diversity compared to both adult male and female *H. contortus*. Data obtained in this study has shown presence of bacterial genera (*Vibrio, Lysinibacillus* and *Anaplasma*) in the *H. contortus* and abomasum that can produce nematicidal volatile compounds that are active against nematodes, with possibility to be manipulated to control populations of GIN parasites within the sheep abomasum. To determine the exact nature of microbiome-*H. contortus* and host-*H. Contortus* microbiome interaction, additional and complementary analyses are necessary to uncover specific molecular mechanisms underlying the nematode-microbiome as a method of controlling nematodes.

## Materials and methods

### Ethical approval

Ethical approval was obtained from the Onderstepoort Veterinary Institute Animal Ethics Committee (AEC) (AEC No: 36.18) and the scientific committee of the Integrated Pest Management subprogram, North-West University. All methods in this study were approved by AEC as well as the scientific committee of the Integrated Pest Management subprogram, North-West University, and performed in accordance with the relevant guidelines and regulations.

### Animals and parasites

The study was conducted using 10 Dohne Merino sheep (Table S1), collected from Wauldby farm (E27° 37′ 32.7"’’ S32° 35′ 07′’) which consist of a flock of over 450 ewes situated in the Stutterheim district in the Eastern Cape Province, South Africa. This farm has a well-documented history of natural *H. contortus* infection.

### Recovery and morphological characterization of gastrointestinal parasites

Naturally infected Dohne Merino sheep were slaughtered under humane conditions. The abomasum was detached from the rest of the GIT and the contents of the abomasum including adult worms were collected^4,33^. The worms were recovered from 7 of the 10 sampled Dohne Merino sheep. The worms were first examined in detail with the assistance of an experienced helminthologist to identify and sexually differentiate worms based on their morphological characters as described by Lichtenfels et al.^20^ and Jacquiet et al.^34^ (Fig. S1). The morphological identification was further confirmed by PCR assay using species specific primers (Table S2).

### Sterilisation of parasitic *H. contortus* surface

The samples were washed with phosphate-buffered saline (PBS), pH 7.4 and incubated in an antibiotic solution (1 mg/ml ampicillin/1 mg/ml gentamicin) for 2 h to kill external bacteria. Thereafter, the worms were washed three times with 4% sodium hypochlorite for 20 s, followed by washing five times with sterile PBS^4,10^. This process was done to remove external and abomasal contaminates on the surface of the adult male and female *H. contortus*. A PCR assay was employed to confirm the absence of microbiome contamination on the GIN surfaces (Fig. S2).

### Microbial DNA isolation

A total of 24 samples were collected from 10 Dohne Merino sheep. Of the 24 samples, 10 of them were abomasum content samples, 7 were pooled adult male and 7 pooled female *H. contortus* worms which were then homogenized and incubated in lysis solution composed of 20 mM Tris·HCl, pH 8.0, 2 mM Sodium EDTA, 1.2% Triton X-100, plus 20 mg/ml Lysozyme at 37 °C for 60 min. Microbial DNA together with GIN parasite DNA was extracted from the pre-processed abomasum contents and the sterile *H. contortus* samples using the QIAamp DNA Microbiome Kit (QIAGEN, Hilden, Germany), as described in the manufacturer’s protocol. The DNA samples were stored at − 20 °C until further processing.

### Amplicon library preparation and sequencing

A region of approximately 470 bp to 500 bp encircling the V3-V4 hypervariable region within the 16S rRNA gene was amplified using a set of commonly used primers for the analysis of the gut microbiome (Table S2). The region of 260 bp was also amplified using species specific primers for confirmation of *H. contortus* (Table S2)^22^. Briefly, each 25 μl of PCR assay reaction contained 5 μl of genomic DNA as the template, 12.5 μl 2× KAPA HiFi HotStart ReadyMix (Kapa Biosystems, United States) and 5 μl of 10 μM of each primer. PCR reactions were carried out in a BIO-RAD T100TM Thermal Cycler (Bio-Rad Laboratories, United Kingdom) using the following protocols: (1) for microbiome, an initial denaturation step performed at 95 °C for 5 min followed by 30 cycles of denaturation (95 °C, 30 s), annealing (56 °C, 30 s) and extension (72 °C, 40 s), and a final elongation of 10 min at 72 °C; (2) for *H. contortus*, an initial denaturation step performed at 95 °C for 5 min followed by 30 cycles of denaturation (95 °C, 30 s), annealing (58 °C, 30 s) and extension (72 °C, 40 s), and a final elongation of 10 min at 72 °C. The PCR amplicon were assessed by gel electrophoresis in 1% agarose gel run at 100 V for 45 min, and the sizes of the products were validated by comparison with a molecular marker. The PCR products were cleaned-up using AMPure XP beads (Beckman Coulter, United States). Amplicon library preparation and sequencing were conducted as per Illumina 16S Metagenomic Sequencing Library Preparation guide. The prepared pooled libraries were then loaded into the Illumina MiSeq sequencing platform.

### Pre-processing and data analysis

De-multiplexed, paired-end fastq reads and a mapping file or metadata file were imported into the Quantitative Insights Into Microbial Ecology 2 program (QIIME2, ver. 2018.6.0, https://qiime2.org/). Sequencing reads were pre-processed, quality filtered and analysed using the Divisive Amplicon Denoising Algorithm 2 (DADA2) software package, wrapped in QIIME2 version 2018.6.0. Resultant feature sequences were summarized and then annotated using the QIIME2 q2- feature-classifier plugin and Silva 132 99% OTUs (full-length, seven-level taxonomy) classifier pre-trained to the full-length Silva database to assign taxonomy to all ribosomal sequence variants.

### Statistical analysis

Alpha and beta-diversity analyses were performed with the q2-diversity plugin wrapped in QIIME2 at a sampling depth of 15,000 sequences. Alpha diversity metrics (Shannon diversity index, Simpson diversity index, Pielou’s measure of species evenness and Faith’s Phylogenetic Diversity) were calculated and statistical significance determined using Kruskal–Wallis tests. The Beta diversity was calculated by Bray–Curtis and Jaccard distances, unweighted and weighted UniFrac metrics. Principal Coordinate analyses (PCoA) was performed based on unweighted and weighted UniFrac distances and visualized with QIIME2 emperor visualizer (ver. 2018.6.0) and the statistical significances were calculated using Permutational Multivariate Analysis Of Variance (PERMANOVA). Additional analysis were performed with in-house python scripts (Python version 3.6. 1, 2017-03-21), KronaTools and RStudio software using phyloseq package (R version 3.5.0, 2018-04-23).

## Supplementary Information

Supplementary Information

## Data Availability

The datasets generated during and/or analysed during the current study are available in the NCBI Sequence Read Archive (SRA) repository, Accession number: PRJNA698475.
