# Supplementary material for: Bacterial profiling of Haemonchus contortus gut microbiome infecting Dohne Merino sheep in South Africa
Source: Sci Rep. 2021 Mar 15;11:5905. doi: 10.1038/s41598-021-85282-7 (PMC7961046; doi:10.1038/s41598-021-85282-7)
Supplement: Supplementary file 1 — Supplementary Information [file 41598_2021_85282_MOESM1_ESM.docx]

*Supplementary Information*

**Bacterial profiling of *Haemonchus contortus* gut microbiome infecting Dohne Merino sheep in South Africa**

T. Mafuna^1,4,6^, P. Soma^2^, A.M. Tsotetsi-Khambule^5^, C.A. Hefer^1,3^, F.C. Muchadeyi ^1^, O.M.M. Thekisoe^6^_,_ R.E. Pierneef^1*^

^1^Agricultural Research Council, Biotechnology Platform, Private Bag X05, Onderstepoort, 0110, South Africa

^2^Agricultural Research Council, Animal Production, Private Bag X2, Irene, 0062, South Africa

^3^AgResearch Ltd, Lincoln Research Center, 1365 Springs Road, Lincoln, 7674, New Zealand

^4^Center for Bioinformatics and Computational Biology, Department of Biochemistry, Genetics and Molecular Biology, University of Pretoria, Lynwood Road, Pretoria, 0001, South Africa

^5^Department of Life and Consumer Sciences, University of South Africa, Florida Campus
Private Bag X6, Florida, 1710, South Africa

^6^Unit for Environmental Sciences and Management, North-West University, Private Bag X6001, Potchefstroom, 2520

Corresponding author: [PierneefR@arc.agric.za](mailto:PierneefR@arc.agric.za), +27 (0)12 529 9356

Keywords: Metabarcoding, Gastrointestinal nematode, *Haemonchus contortus*, metagenomics, gut microbiome

**Supplementary Materials**

**Table S1.** Percentage of total number of adult male and female *H. contortus* species identified in the present study.

| Animals ID | Age | No of *H. c* | Observed male *H. c* | %Male | Observed female *H. c* | %Female |
| --- | --- | --- | --- | --- | --- | --- |
| B1.187 | 7 | 26 | 9 | 35% | 17 | 65% |
| B2.204 | 6 | - | - | - | - | - |
| B1.098 | 7 | - | - | - | - | - |
| B1.075 | 7 | 42 | 11 | 26% | 31 | 73% |
| B1.219 | 7 | 50 | 21 | 42% | 29 | 58% |
| B0.515 | 8 | 50 | 23 | 46% | 27 | 54% |
| B9.181 | 9 | 50 | 20 | 40% | 30 | 60% |
| B9.233 | 9 | 28 | 7 | 25% | 21 | 75% |
| B9.053 | 9 | - | - | - | - | - |
| B1.168 | 7 | 20 | 6 | 30% | 14 | 70% |
| Total |  | **266** | **97** |  | **169** |  |
| Overall % |  |  | | **36.5%** |  | **63.5%** |

**Table S2.** Summary of primers used in the current study

| Primer pairs | Primer sequence (5′−3′) | Targeted regions | Reference |
| --- | --- | --- | --- |
| 338F | 5′-CCTACGGGNGGCWGCAG-3′ | V3-V4 region | Klindworth *et al.,* 2013 |
| 806R | 5′-GACTACHVGGGTATCTAATCC-3′ |  |  |
| HcBotuF1 | 5′-TGTCGAACACGAAACTCGTC-3′ | *H. contortus* | Amarante *et al*., 2017 |
| HcBotuR2 | 5′-TGTGTCTCTACCGCCCGAGT-3′. |  |  |

**Table S3.** Overview of beta diversity for the abomasum content, adult male and female *H.* *contortus* microbiome

| Pairwise distances | Permutations | Pseudo-*F* | P-value | q-value |
| --- | --- | --- | --- | --- |
| Abomasum to Male gut | 999 | 14.711808 | 0.001 | 0.0015 |
| Abomasum to Female gut | 999 | 43.357699 | 0.001 | 0.0015 |
| Male to Female | 999 | 2.850805 | 0.029 | 0.0290 |

*P*-values and pseudo-*F* were calculated using PERMANOVA test (Pseudo-*F*). The level of significance was determined at *P* < 0.05.


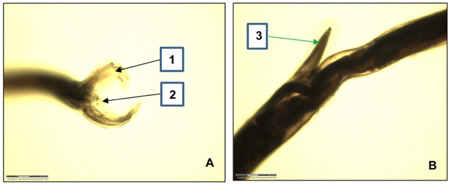


**Figure S1.** Picture depicting *H. contortus* characters (**A**). Male bursa and (**B**). Female vulva flap. The black arrows are pointing to the male copulatory bursa (**1**) and spicules (**2**), the green arrow is pointing to the female vulval flap (**3**).


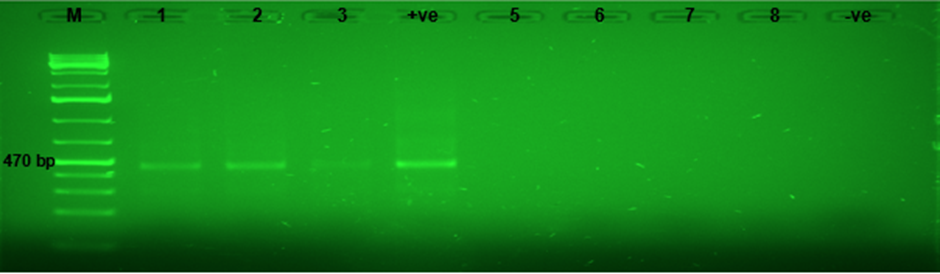


**Figure S2.** Gel electrophoresis image from 1.5% Agarose gel stained with ethidium

112 bromide showing washed and unwashed *H. contortus* PCR amplicons at 470 bp. (M) as 100 bp Marker (Generuler DNA ladder, Thermo Fisher Scientific, South Africa) . (+ve) as positive control. (–ve) double distilled water used as negative control. Line (1-4) unwashed. Line (5-9) washed *H. contortus.*


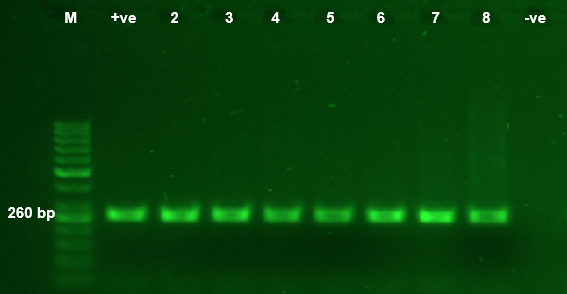


**Figure S3.** Gel electrophoresis image from 1.5% Agarose gel stained with ethidium

120 bromide depicting showing *H. contortus* PCR amplicons at 260 bp. (M) as 50 bp Marker (GeneRuler DNA ladder, Thermo Fisher Scientific, South Africa). Line (1-8) *H. contortus*. (+ve) *H. contortus* as positive control. (-ve) double distilled water used as negative control.


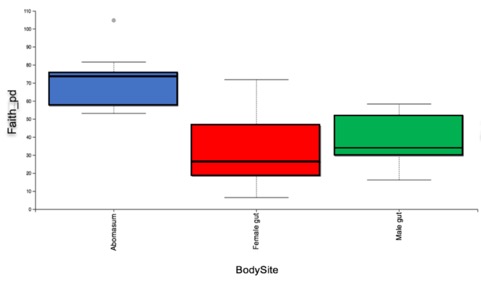


**Figure S4.** Alpha diversity analysis the abomasum content, male and female *H. contortus* microbiome at 97% identity. Alpha diversity matrices Faith_pd. Data represent average values from pooled populations.

**
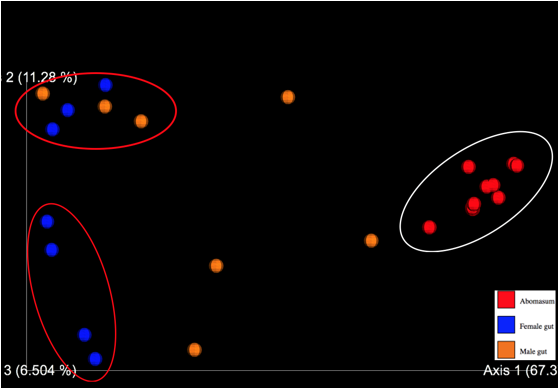
**

**Figure S5**. Principal coordinate analysis (PCoA) based on weighted UniFrac distances (Considers taxa abundances). Data represent average values from pooled populations.

**Figure S6**. Bar chart showing the relative OTUs abundances of the abomasum content, adult female and male *H. contortus*. Data represent average values from pooled populations.

**Table S4**. Total of 660 OTUs were detected in the abomasum, adult male and female *H. contortus*.

| Hits | Kingdom | Phylum | Class | Order | Family | Genus | Species |
| --- | --- | --- | --- | --- | --- | --- | --- |
| 32 | Bacteria | Planctomycetes | Planctomycetacia | Pirellulales | Pirellulaceae | Pirellula | uncultured Planctomycetales bacterium |
| 138 | Bacteria | Proteobacteria | Gammaproteobacteria | Enterobacteriales | Enterobacteriaceae | Pantoea |  |
| 24 | Bacteria | Actinobacteria | Actinobacteria | Actinomycetales | Actinomycetaceae | uncultured | uncultured rumen bacterium |
| 16 | Bacteria | Actinobacteria | Actinobacteria | Corynebacteriales | Nocardiaceae | Nocardia |  |
| 219 | Bacteria | Proteobacteria | Alphaproteobacteria | Rhizobiales | Rhizobiaceae |  |  |
| 177 | Bacteria | Firmicutes | Clostridia | Clostridiales | Ruminococcaceae | Ruminococcaceae UCG-014 | gut metagenome |
| 19 | Bacteria | Bacteroidetes | Bacteroidia | Bacteroidales | p-2534-18B5 gut group | uncultured bacterium | uncultured bacterium |
| 41 | Bacteria | Actinobacteria | Actinobacteria | Micrococcales | Microbacteriaceae |  |  |
| 729 | Bacteria | Actinobacteria | Coriobacteriia | Coriobacteriales | Coriobacteriales Incertae Sedis | Raoultibacter | uncultured bacterium |
| 15 | Bacteria | Firmicutes | Bacilli | Bacillales | Planococcaceae |  |  |
| 50 | Bacteria | Tenericutes | Mollicutes | Mycoplasmatales | Mycoplasmataceae | Mycoplasma | uncultured rumen bacterium |
| 71 | Bacteria | Firmicutes | Clostridia | Clostridiales | Family XI | Finegoldia | Finegoldia magna |
| 677 | Bacteria | Bacteroidetes | Bacteroidia | Bacteroidales | Bacteroidetes BD2-2 | uncultured rumen bacterium | uncultured rumen bacterium |
| 11 | Bacteria | Acidobacteria | Acidobacteriia | Subgroup 2 |  |  |  |
| 202 | Bacteria | Proteobacteria | Deltaproteobacteria | Bradymonadales | uncultured rumen bacterium | uncultured rumen bacterium | uncultured rumen bacterium |
| 569 | Bacteria | Bacteroidetes | Bacteroidia | Bacteroidales | F082 | Bacteroidales bacterium Bact_22 | Bacteroidales bacterium Bact_22 |
| 8 | Bacteria | Firmicutes | Clostridia | Clostridiales | Lachnospiraceae | Anaerocolumna |  |
| 4633 | Bacteria | Bacteroidetes | Bacteroidia | Bacteroidales | Rikenellaceae | U29-B03 | uncultured rumen bacterium |
| 55 | Bacteria | Actinobacteria | Actinobacteria | Micromonosporales | Micromonosporaceae |  |  |
| 5 | Bacteria | Bacteroidetes | Bacteroidia | Bacteroidales | Prevotellaceae | Prevotella 7 |  |
| 13 | Bacteria | Proteobacteria | Gammaproteobacteria | Alteromonadales | Idiomarinaceae | Idiomarina |  |
| 101 | Bacteria | Firmicutes | Clostridia | Clostridiales | Ruminococcaceae | Ruminococcaceae UCG-010 | uncultured rumen bacterium 4C0d-9 |
| 1055 | Bacteria | Firmicutes | Clostridia | Clostridiales | Lachnospiraceae | Coprococcus 1 | uncultured rumen bacterium |
| 2572 | Bacteria | Firmicutes | Clostridia | Clostridiales | Lachnospiraceae | Butyrivibrio 2 |  |
| 18 | Bacteria | Firmicutes | Clostridia | Clostridiales | Clostridiales vadinBB60 group | uncultured Firmicutes bacterium | uncultured Firmicutes bacterium |
| 461 | Bacteria | Spirochaetes | Spirochaetia | Spirochaetales | Spirochaetaceae | Treponema 2 | Treponema porcinum |
| 10 | Bacteria | Bacteroidetes | Bacteroidia | Bacteroidales | Dysgonomonadaceae | Dysgonomonas | uncultured Dysgonomonas sp. |
| 25493 | Bacteria | Planctomycetes | Planctomycetacia | Pirellulales | Pirellulaceae | p-1088-a5 gut group | uncultured rumen bacterium |
| 76 | Bacteria | Bacteroidetes | Bacteroidia | Bacteroidales | Prevotellaceae | Prevotella 1 | unidentified rumen bacterium 12-12 |
| 111 | Bacteria | Firmicutes | Bacilli | Bacillales |  |  |  |
| 210 | Bacteria | Proteobacteria | Gammaproteobacteria | Betaproteobacteriales | Burkholderiaceae |  |  |
| 592 | Bacteria | Firmicutes | Clostridia | Clostridiales | Lachnospiraceae | Shuttleworthia | uncultured Lachnospiraceae bacterium |
| 24675 | Bacteria | Patescibacteria | Saccharimonadia | Saccharimonadales | Saccharimonadaceae | Candidatus Saccharimonas | uncultured rumen bacterium |
| 2933 | Archaea | Euryarchaeota | Methanobacteria | Methanobacteriales | Methanobacteriaceae | Methanosphaera |  |
| 31 | Bacteria | Firmicutes | Clostridia | Clostridiales | Clostridiaceae 3 | Clostridiisalibacter |  |
| 9 | Bacteria | Firmicutes | Clostridia | Clostridiales | Ruminococcaceae | Ruminococcaceae UCG-009 | uncultured Firmicutes bacterium |
| 39 | Bacteria | Bacteroidetes | Bacteroidia | Flavobacteriales | Flavobacteriaceae | Myroides | Myroides odoratimimus |
| 469 | Bacteria | Actinobacteria | Actinobacteria | Actinomycetales | Actinomycetaceae | Actinomyces |  |
| 579 | Bacteria | Firmicutes | Clostridia | Clostridiales | Lachnospiraceae | Lachnospiraceae UCG-002 |  |
| 118 | Bacteria | Bacteroidetes | Bacteroidia | Bacteroidales | Marinifilaceae | uncultured | uncultured rumen bacterium |
| 1082 | Bacteria | Bacteroidetes | Bacteroidia | Bacteroidales | p-251-o5 | uncultured rumen bacterium | uncultured rumen bacterium |
| 64 | Bacteria | Bacteroidetes | Bacteroidia | Bacteroidales | Rikenellaceae | hoa5-07d05 gut group | uncultured rumen bacterium |
| 15 | Bacteria | Firmicutes | Bacilli | Bacillales | Staphylococcaceae | Staphylococcus | Staphylococcus aureus |
| 3942 | Bacteria | Firmicutes | Clostridia | Clostridiales | Ruminococcaceae | Saccharofermentans |  |
| 290 | Bacteria | Firmicutes | Clostridia | Clostridiales | Ruminococcaceae | Ruminococcaceae NK4A214 group | uncultured organism |
| 11 | Bacteria | Firmicutes | Clostridia | Clostridiales | Syntrophomonadaceae |  |  |
| 2746 | Bacteria | Firmicutes | Clostridia | Clostridiales | Christensenellaceae | Christensenellaceae R-7 group | bacterium AC2043 |
| 33 | Bacteria | Armatimonadetes | Armatimonadia | Armatimonadales |  |  |  |
| 132 | Bacteria | Bacteroidetes | Bacteroidia | Bacteroidales | p-251-o5 | uncultured Bacteroidales bacterium | uncultured Bacteroidales bacterium |
| 726 | Bacteria | Bacteroidetes | Bacteroidia | Bacteroidales | Prevotellaceae | Prevotellaceae UCG-004 | uncultured rumen bacterium 4C0d-8 |
| 646 | Bacteria | Elusimicrobia | Endomicrobia | Endomicrobiales | Endomicrobiaceae | Candidatus Endomicrobium | uncultured rumen bacterium |
| 1838 | Bacteria | Firmicutes | Erysipelotrichia | Erysipelotrichales | Erysipelotrichaceae | Solobacterium | uncultured rumen bacterium |
| 71 | Bacteria | Proteobacteria | Alphaproteobacteria | Rhodobacterales | Rhodobacteraceae | Paracoccus |  |
| 2114 | Bacteria | Firmicutes | Clostridia | Clostridiales | Lachnospiraceae | Syntrophococcus | uncultured rumen bacterium |
| 847 | Bacteria | Proteobacteria | Deltaproteobacteria | Desulfovibrionales | Desulfovibrionaceae | Desulfovibrio |  |
| 1666 | Bacteria | Bacteroidetes | Bacteroidia | Bacteroidales | Bacteroidales BS11 gut group | uncultured rumen bacterium | uncultured rumen bacterium |
| 1509 | Bacteria | Actinobacteria | Actinobacteria | Propionibacteriales | Propionibacteriaceae | Cutibacterium |  |
| 165 | Bacteria | Bacteroidetes | Bacteroidia | Bacteroidales | Prevotellaceae | Prevotella 1 | bacterium XPB4001 |
| 3 | Bacteria | Firmicutes | Negativicutes | Selenomonadales | Veillonellaceae | Dialister | unidentified |
| 97 | Bacteria | Actinobacteria | Coriobacteriia |  |  |  |  |
| 69275 | Bacteria | Proteobacteria | Gammaproteobacteria | Vibrionales | Vibrionaceae | Vibrio |  |
| 380 | Bacteria | Bacteroidetes | Bacteroidia | Bacteroidales | F082 | bacterium P201 | bacterium P201 |
| 11 | Bacteria | Proteobacteria | Alphaproteobacteria | Sphingomonadales | Sphingomonadaceae | Altererythrobacter |  |
| 39 | Bacteria | Cyanobacteria | Oxyphotobacteria | Chloroplast | Medicago truncatula (barrel medic) | Medicago truncatula (barrel medic) | Medicago truncatula (barrel medic) |
| 1177 | Bacteria | Bacteroidetes | Bacteroidia | Bacteroidales | Prevotellaceae | Prevotellaceae YAB2003 group | uncultured rumen bacterium |
| 6 | Bacteria | Firmicutes | Clostridia | Clostridiales | Clostridiaceae 1 | Proteiniclasticum | uncultured Clostridium sp. |
| 2418 | Bacteria | Firmicutes | Clostridia | Clostridiales | Family XIII | Anaerovorax | uncultured rumen bacterium |
| 31 | Bacteria | Proteobacteria | Alphaproteobacteria | Sphingomonadales | Sphingomonadaceae | Sandaracinobacter | uncultured bacterium |
| 11 | Bacteria | Actinobacteria | Actinobacteria | Micrococcales | Micrococcaceae | Paenarthrobacter |  |
| 274 | Bacteria | Firmicutes | Clostridia | Clostridiales | Ruminococcaceae | [Eubacterium] coprostanoligenes group | gut metagenome |
| 6 | Bacteria | Actinobacteria | Rubrobacteria | Rubrobacterales | Rubrobacteriaceae | Rubrobacter | uncultured organism |
| 4 | Bacteria | Proteobacteria | Alphaproteobacteria | Tistrellales | Geminicoccaceae | Geminicoccus | uncultured bacterium |
| 38093 | Bacteria | Bacteroidetes | Bacteroidia | Bacteroidales | Prevotellaceae |  |  |
| 1679 | Bacteria | Firmicutes | Clostridia | Clostridiales | Lachnospiraceae | Acetitomaculum | uncultured rumen bacterium |
| 373 | Bacteria | Proteobacteria | Deltaproteobacteria | Desulfobacterales | Desulfobulbaceae | Desulfobulbus | uncultured rumen bacterium |
| 2705 | Bacteria | Spirochaetes | Spirochaetia | Spirochaetales | Spirochaetaceae | Treponema 2 | uncultured Spirochaetaceae bacterium |
| 3 | Bacteria | Chlamydiae | Chlamydiae | Chlamydiales | Parachlamydiaceae | Neochlamydia | metagenome |
| 4329 | Bacteria | Bacteroidetes | Bacteroidia | Bacteroidales | Rikenellaceae | SP3-e08 | uncultured bacterium |
| 807 | Bacteria | Firmicutes | Clostridia | Clostridiales | Eubacteriaceae | Pseudoramibacter | uncultured bacterium |
| 57 | Bacteria | Tenericutes | Mollicutes | Anaeroplasmatales | Anaeroplasmataceae | Anaeroplasma |  |
| 265 | Bacteria | Firmicutes | Erysipelotrichia | Erysipelotrichales | Erysipelotrichaceae | Erysipelotrichaceae UCG-004 | uncultured rumen bacterium |
| 26 | Bacteria | Proteobacteria | Alphaproteobacteria | Rhizobiales | Beijerinckiaceae |  |  |
| 1129 | Bacteria | Firmicutes | Bacilli | Lactobacillales | Enterococcaceae | Enterococcus |  |
| 9 | Bacteria | Firmicutes | Clostridia | Clostridiales | Lachnospiraceae | [Eubacterium] oxidoreducens group | Eubacterium oxidoreducens |
| 2967 | Bacteria | Firmicutes | Clostridia | Clostridiales | Family XIII | Family XIII AD3011 group | uncultured organism |
| 792 | Bacteria | Firmicutes | Clostridia | Clostridiales | Ruminococcaceae | [Eubacterium] coprostanoligenes group | uncultured Clostridia bacterium |
| 72487 | Bacteria | Firmicutes | Clostridia | Clostridiales | Lachnospiraceae |  |  |
| 11 | Bacteria | Proteobacteria | Alphaproteobacteria | Rhizobiales | Methyloligellaceae | uncultured |  |
| 4 | Bacteria | Tenericutes | Mollicutes | Izimaplasmatales | Izimaplasmataceae | unidentified rumen bacterium RF3 | unidentified rumen bacterium RF3 |
| 8 | Bacteria | Actinobacteria | Thermoleophilia | Solirubrobacterales | Solirubrobacteraceae | Patulibacter | uncultured bacterium |
| 92 | Bacteria | Firmicutes | Clostridia | Clostridiales | Family XI | Peptoniphilus | uncultured organism |
| 11 | Eukaryota | Opisthokonta | Holozoa | Metazoa (Animalia) | D_7Chromadorea | D_8Rhabditida | D_9Haemonchus contortus (barber pole worm) |
| 949 | Bacteria | Lentisphaerae | Oligosphaeria | Oligosphaerales | Oligosphaeraceae | Z20 | uncultured rumen bacterium |
| 162 | Bacteria | Actinobacteria | Actinobacteria | Corynebacteriales | Nocardiaceae | Rhodococcus |  |
| 407 | Bacteria | Firmicutes | Clostridia | Clostridiales | Ruminococcaceae | Ruminococcaceae UCG-002 | gut metagenome |
| 20 | Bacteria | Firmicutes | Clostridia | Clostridiales | Eubacteriaceae |  |  |
| 13131 | Bacteria | Firmicutes | Clostridia | Clostridiales | Lachnospiraceae | Lachnospiraceae NK3A20 group |  |
| 38 | Bacteria | Bacteroidetes | Bacteroidia | Sphingobacteriales | Sphingobacteriaceae | Sphingobacterium |  |
| 581 | Bacteria | Bacteroidetes | Bacteroidia | Bacteroidales | Prevotellaceae | Prevotella 1 | Prevotella sp. BP1-56 |
| 73 | Bacteria | Planctomycetes | Phycisphaerae | Tepidisphaerales | WD2101 soil group |  |  |
| 665 | Bacteria | Bacteroidetes | Bacteroidia | Bacteroidales | Bacteroidales BS11 gut group |  |  |
| 19 | Bacteria | Bacteroidetes | Bacteroidia | Flavobacteriales | Flavobacteriaceae | Salinimicrobium | uncultured Salegentibacter sp. |
| 634 | Bacteria | Proteobacteria | Deltaproteobacteria | Oligoflexales | 0319-6G20 | uncultured rumen bacterium | uncultured rumen bacterium |
| 16 | Bacteria | Actinobacteria | Thermoleophilia | Gaiellales | uncultured |  |  |
| 16 | Bacteria | Bacteroidetes | Bacteroidia | Sphingobacteriales | Sphingobacteriaceae | Sphingobacterium | Sphingobacterium sp. |
| 22 | Bacteria | Bacteroidetes | Bacteroidia | Bacteroidales | Rikenellaceae | Rikenellaceae RC9 gut group | uncultured Bacteroidales bacterium |
| 72 | Bacteria | Firmicutes | Erysipelotrichia | Erysipelotrichales | Erysipelotrichaceae | Sharpea | uncultured bacterium |
| 147 | Bacteria | Firmicutes | Clostridia | Clostridiales | Lachnospiraceae | Acetitomaculum |  |
| 4232 | Bacteria | Bacteroidetes | Bacteroidia | Bacteroidales | Marinilabiliaceae | uncultured | uncultured rumen bacterium |
| 1218 | Bacteria | Firmicutes | Clostridia | Clostridiales | Ruminococcaceae | Ruminococcaceae UCG-013 | uncultured rumen bacterium |
| 8 | Bacteria | Firmicutes | Bacilli | Lactobacillales | Carnobacteriaceae | Alloiococcus | uncultured bacterium |
| 3207 | Bacteria | Firmicutes | Erysipelotrichia | Erysipelotrichales | Erysipelotrichaceae | uncultured | uncultured rumen bacterium |
| 2300 | Bacteria | Firmicutes | Clostridia | Clostridiales | Lachnospiraceae | uncultured | uncultured Clostridium sp. |
| 10 | Bacteria | Verrucomicrobia | Verrucomicrobiae | Verrucomicrobiales | Akkermansiaceae | Akkermansia | uncultured Verrucomicrobia bacterium |
| 710 | Bacteria | Proteobacteria | Deltaproteobacteria | Desulfovibrionales | Desulfovibrionaceae | Mailhella |  |
| 551 | Bacteria | Spirochaetes | Spirochaetia | Spirochaetales | Spirochaetaceae | Sphaerochaeta | uncultured Spirochaeta sp. |
| 447 | Bacteria | Firmicutes | Clostridia | Clostridiales | Lachnospiraceae | Butyrivibrio 2 | Butyrivibrio fibrisolvens |
| 6 | Bacteria | Fusobacteria | Fusobacteriia | Fusobacteriales | Leptotrichiaceae | Hypnocyclicus | uncultured bacterium |
| 1995 | Bacteria | Firmicutes | Bacilli | Lactobacillales | Carnobacteriaceae |  |  |
| 23 | Bacteria | Bacteroidetes | Bacteroidia | Sphingobacteriales | Sphingobacteriaceae | Sphingobacterium | Sphingobacterium spiritivorum |
| 2023 | Bacteria | Firmicutes | Clostridia | Clostridiales | Ruminococcaceae | [Eubacterium] coprostanoligenes group | uncultured Ruminococcaceae bacterium |
| 1606 | Bacteria | Patescibacteria | Gracilibacteria | Absconditabacteriales (SR1) | uncultured rumen bacterium | uncultured rumen bacterium | uncultured rumen bacterium |
| 1013 | Bacteria | Planctomycetes | Planctomycetacia | Pirellulales | Pirellulaceae | Pirellula | uncultured rumen bacterium |
| 9 | Bacteria | Tenericutes | Mollicutes | Izimaplasmatales |  |  |  |
| 86 | Bacteria | Spirochaetes | Spirochaetia | Spirochaetales | Spirochaetaceae |  |  |
| 5 | Bacteria | Lentisphaerae | Oligosphaeria | Oligosphaerales | Oligosphaeraceae | horsej-a03 | uncultured bacterium |
| 7 | Bacteria | Bacteroidetes | Bacteroidia | Cytophagales | Hymenobacteraceae | Adhaeribacter | uncultured Bacteroidetes bacterium |
| 54 | Bacteria | Firmicutes | Clostridia | Clostridiales | Ruminococcaceae | Papillibacter |  |
| 755 | Bacteria | Actinobacteria | Coriobacteriia | Coriobacteriales | uncultured |  |  |
| 30 | Bacteria | Firmicutes | Bacilli | Bacillales | Planococcaceae | Sporosarcina |  |
| 2895 | Bacteria | Firmicutes | Clostridia | Clostridiales | Family XIII | Family XIII AD3011 group |  |
| 26 | Bacteria | Firmicutes | Clostridia | Clostridiales | Ruminococcaceae | Ruminococcaceae UCG-007 | uncultured rumen bacterium |
| 2127 | Bacteria | Firmicutes | Clostridia | Clostridiales | Lachnospiraceae | Lachnospiraceae ND3007 group | uncultured Lachnospiraceae bacterium |
| 42 | Bacteria | Actinobacteria | Actinobacteria | Pseudonocardiales | Pseudonocardiaceae | Pseudonocardia |  |
| 12 | Bacteria | Actinobacteria | Actinobacteria | Micrococcales | Dermabacteraceae | Brachybacterium | Brachybacterium paraconglomeratum |
| 45 | Bacteria | Proteobacteria | Deltaproteobacteria | Desulfovibrionales | Desulfovibrionaceae |  |  |
| 1589 | Bacteria | Proteobacteria | Gammaproteobacteria | Pseudomonadales | Moraxellaceae | Enhydrobacter |  |
| 9 | Bacteria | Firmicutes | Clostridia | Clostridiales | Family XI | Anaerococcus |  |
| 2433 | Bacteria | Firmicutes | Clostridia | Clostridiales | Christensenellaceae | Christensenellaceae R-7 group | uncultured prokaryote |
| 2691 | Bacteria | Chloroflexi | Anaerolineae | Anaerolineales | Anaerolineaceae | Flexilinea | uncultured rumen bacterium |
| 7 | Bacteria | Actinobacteria | Actinobacteria | Corynebacteriales | Dietziaceae | Dietzia | Dietzia maris |
| 79 | Bacteria | Bacteroidetes | Bacteroidia | Flavobacteriales | Flavobacteriaceae | Mesonia |  |
| 1990 | Bacteria | Firmicutes | Clostridia | Clostridiales | Ruminococcaceae | Papillibacter | uncultured rumen bacterium |
| 45 | Bacteria | Firmicutes | Clostridia | Clostridiales | Ruminococcaceae | uncultured | uncultured rumen bacterium 4C0d-17 |
| 6467 | Bacteria | Firmicutes | Clostridia | Clostridiales | Clostridiaceae 1 | Clostridium sensu stricto 1 | Clostridium sp. ND2 |
| 764 | Bacteria | Firmicutes | Clostridia | Clostridiales | Lachnospiraceae | [Eubacterium] ruminantium group | uncultured rumen bacterium |
| 157 | Bacteria | Firmicutes | Clostridia | Clostridiales | Lachnospiraceae | Lachnospiraceae ND3007 group |  |
| 135 | Bacteria | Proteobacteria | Alphaproteobacteria | Rhizobiales | Rhizobiaceae | Pseudochrobactrum | Pseudochrobactrum saccharolyticum |
| 5223 | Bacteria | Firmicutes |  |  |  |  |  |
| 171 | Bacteria | Firmicutes | Clostridia | Clostridiales | Lachnospiraceae | Oribacterium | uncultured Lachnospiraceae bacterium |
| 76 | Bacteria | Actinobacteria | Actinobacteria | Propionibacteriales | Nocardioidaceae | Nocardioides |  |
| 38 | Bacteria | Proteobacteria | Alphaproteobacteria | Rhizobiales | Beijerinckiaceae | Microvirga |  |
| 915 | Bacteria | Actinobacteria | Coriobacteriia | Coriobacteriales | Atopobiaceae | Atopobium |  |
| 140 | Bacteria | Firmicutes | Clostridia | Clostridiales | Lachnospiraceae | Lachnoclostridium 1 | uncultured bacterium |
| 58 | Bacteria | Bacteroidetes | Bacteroidia | Bacteroidales | Rikenellaceae | Rikenellaceae RC9 gut group | unidentified rumen bacterium RF36 |
| 2282 | Bacteria | Proteobacteria | Gammaproteobacteria | Xanthomonadales | Xanthomonadaceae | Stenotrophomonas |  |
| 309 | Bacteria | Epsilonbacteraeota | Campylobacteria | Campylobacterales | Campylobacteraceae | Campylobacter |  |
| 317 | Bacteria | Firmicutes | Clostridia | Clostridiales | Lachnospiraceae | Lachnospiraceae FE2018 group |  |
| 33 | Bacteria | Actinobacteria | Coriobacteriia | Coriobacteriales | Coriobacteriales Incertae Sedis | uncultured |  |
| 88 | Bacteria | Firmicutes | Clostridia | Clostridiales | Ruminococcaceae | CAG-352 | uncultured rumen bacterium |
| 15 | Bacteria | Firmicutes | Clostridia | Clostridiales | Ruminococcaceae | Ruminococcus 1 | uncultured organism |
| 2679 | Bacteria | Firmicutes | Negativicutes | Selenomonadales | Veillonellaceae | Selenomonas 1 |  |
| 7010 | Bacteria | Spirochaetes | Spirochaetia | Spirochaetales | Spirochaetaceae | Treponema 2 |  |
| 22 | Bacteria | Firmicutes | Clostridia | Clostridiales | Christensenellaceae | uncultured | uncultured rumen bacterium |
| 1784 | Bacteria | Firmicutes | Clostridia | Clostridiales | Ruminococcaceae | Ruminococcaceae UCG-005 | metagenome |
| 16 | Bacteria | Actinobacteria | Actinobacteria | Micrococcales | Micrococcaceae | Rothia |  |
| 121 | Bacteria | Actinobacteria | Coriobacteriia | Coriobacteriales | Coriobacteriales Incertae Sedis | Phoenicibacter | uncultured bacterium |
| 51712 | Bacteria | Firmicutes | Negativicutes | Selenomonadales | Acidaminococcaceae | Succiniclasticum | uncultured Veillonellaceae bacterium |
| 412 | Bacteria | Firmicutes | Clostridia | Clostridiales | Lachnospiraceae | Lachnoclostridium 12 | uncultured rumen bacterium |
| 44 | Bacteria | Actinobacteria | Coriobacteriia | Coriobacteriales | Atopobiaceae | uncultured | uncultured bacterium |
| 143 | Bacteria | Proteobacteria | Gammaproteobacteria | Alteromonadales | Idiomarinaceae | Aliidiomarina | Aliidiomarina sp. |
| 112 | Bacteria | Firmicutes | Bacilli | Lactobacillales | Streptococcaceae | Lactococcus |  |
| 61 | Bacteria | Firmicutes | Clostridia | Clostridiales | Family XI | Anaerococcus | uncultured Anaerococcus sp. |
| 318 | Bacteria | Actinobacteria | Actinobacteria | Corynebacteriales | Corynebacteriaceae | Corynebacterium 1 |  |
| 105 | Bacteria | Bacteroidetes | Bacteroidia | Bacteroidales | Bacteroidales BS11 gut group | uncultured bacterium | uncultured bacterium |
| 4 | Bacteria | Proteobacteria | Alphaproteobacteria | Acetobacterales | Acetobacteraceae |  |  |
| 8 | Bacteria | Firmicutes | Clostridia | Clostridiales | Lachnospiraceae | Catonella |  |
| 49 | Bacteria | Actinobacteria | Coriobacteriia | Coriobacteriales | uncultured | uncultured Coriobacteriaceae bacterium | uncultured Coriobacteriaceae bacterium |
| 12 | Bacteria | Actinobacteria | Coriobacteriia | Coriobacteriales | Eggerthellaceae | Slackia | unidentified |
| 28 | Bacteria | Proteobacteria | Gammaproteobacteria | Betaproteobacteriales | Burkholderiaceae | Lautropia | metagenome |
| 28368 | Bacteria | Firmicutes | Clostridia | Clostridiales | Ruminococcaceae | Ruminococcaceae UCG-011 | uncultured rumen bacterium |
| 14 | Bacteria | Firmicutes | Bacilli | Lactobacillales | Streptococcaceae | Streptococcus | Streptococcus parauberis |
| 459 | Bacteria | Firmicutes | Clostridia | Clostridiales | Christensenellaceae |  |  |
| 4 | Bacteria | Actinobacteria | MB-A2-108 | metagenome | metagenome | metagenome | metagenome |
| 40 | Bacteria | Bacteroidetes | Bacteroidia | Bacteroidales | Prevotellaceae | Prevotella | Chlamydia trachomatis |
| 24 | Bacteria | Actinobacteria | Acidimicrobiia | Microtrichales | uncultured | uncultured Actinomycetales bacterium | uncultured Actinomycetales bacterium |
| 29 | Bacteria | Epsilonbacteraeota | Campylobacteria | Campylobacterales | Arcobacteraceae | Arcobacter |  |
| 9 | Bacteria | Actinobacteria | Actinobacteria | Corynebacteriales | Corynebacteriaceae | Turicella |  |
| 8 | Archaea | Euryarchaeota | Thermoplasmata | Methanomassiliicoccales | Methanomethylophilaceae | Candidatus Methanomethylophilus |  |
| 24 | Bacteria | Firmicutes | Bacilli | Bacillales | Family X | Thermicanus |  |
| 1962 | Bacteria | Firmicutes | Clostridia | Clostridiales | Ruminococcaceae | Saccharofermentans | uncultured rumen bacterium 5C0d-4 |
| 89 | Bacteria | Lentisphaerae | Oligosphaeria | Oligosphaerales | Oligosphaeraceae | Z20 |  |
| 1664 | Bacteria | Firmicutes | Negativicutes | Selenomonadales | Veillonellaceae | Selenomonas 1 | Selenomonas ruminantium AB3002 |
| 23 | Bacteria | Fusobacteria | Fusobacteriia | Fusobacteriales | Fusobacteriaceae | Fusobacterium | Fusobacterium necrophorum subsp. necrophorum |
| 12 | Bacteria | Firmicutes | Bacilli | Lactobacillales | uncultured | uncultured bacterium | uncultured bacterium |
| 4367 | Bacteria | Bacteroidetes | Bacteroidia | Bacteroidales | Rikenellaceae | Rikenellaceae RC9 gut group | uncultured Rikenella sp. |
| 223 | Bacteria | Firmicutes | Clostridia | Clostridiales | Ruminococcaceae | Ruminiclostridium 9 | uncultured Clostridia bacterium |
| 163 | Bacteria | Actinobacteria | Coriobacteriia | Coriobacteriales | Eggerthellaceae | Parvibacter | uncultured bacterium |
| 139 | Bacteria | Firmicutes | Clostridia | Clostridiales | Lachnospiraceae | Lachnospiraceae ND3007 group | unidentified rumen bacterium RC20 |
| 58 | Bacteria | Firmicutes | Clostridia | Clostridiales | Ruminococcaceae | CAG-352 | uncultured bacterium |
| 27 | Bacteria | Proteobacteria | Gammaproteobacteria | Alteromonadales | Alteromonadaceae | Rheinheimera |  |
| 12165 | Bacteria | Firmicutes | Clostridia | Clostridiales | Lachnospiraceae | [Ruminococcus] gauvreauii group | uncultured rumen bacterium |
| 427 | Bacteria | Proteobacteria | Alphaproteobacteria | Sphingomonadales | Sphingomonadaceae | Sphingomonas |  |
| 4 | Bacteria | Bacteroidetes | Bacteroidia | Flavobacteriales | Weeksellaceae | Cloacibacterium |  |
| 303 | Bacteria | Proteobacteria | Gammaproteobacteria | Betaproteobacteriales | Rhodocyclaceae |  |  |
| 1002 | Bacteria | Bacteroidetes | Bacteroidia | Bacteroidales | F082 |  |  |
| 21 | Bacteria | Proteobacteria | Gammaproteobacteria | Xanthomonadales | Xanthomonadaceae | Pseudoxanthomonas |  |
| 1414 | Bacteria | Bacteroidetes | Bacteroidia | Flavobacteriales | Weeksellaceae | Chryseobacterium |  |
| 30 | Bacteria | Firmicutes | Clostridia | Clostridiales | Lachnospiraceae | probable genus 10 |  |
| 6003 | Bacteria | Proteobacteria | Gammaproteobacteria | Alteromonadales | Alteromonadaceae | Alishewanella | uncultured bacterium |
| 208 | Bacteria | Firmicutes | Clostridia | Clostridiales | Family XIII | [Eubacterium] nodatum group |  |
| 353 | Bacteria | Firmicutes | Erysipelotrichia | Erysipelotrichales | Erysipelotrichaceae | Erysipelotrichaceae UCG-009 |  |
| 353 | Bacteria | Firmicutes | Clostridia | Clostridiales | Lachnospiraceae | Butyrivibrio 2 | uncultured Lachnospiraceae bacterium |
| 26 | Bacteria | Actinobacteria | Actinobacteria | Micrococcales | Micrococcaceae | Kocuria |  |
| 7 | Bacteria | Chloroflexi | KD4-96 | uncultured Chloroflexi bacterium | uncultured Chloroflexi bacterium | uncultured Chloroflexi bacterium | uncultured Chloroflexi bacterium |
| 43 | Bacteria | Firmicutes | Clostridia | Clostridiales | Ruminococcaceae | Oscillospira | Oscillospira guilliermondii |
| 305 | Bacteria | Firmicutes | Clostridia | Clostridiales | Lachnospiraceae | Blautia | uncultured rumen bacterium |
| 7316 | Bacteria | Actinobacteria | Coriobacteriia | Coriobacteriales | Eggerthellaceae | DNF00809 | uncultured bacterium |
| 13 | Bacteria | Spirochaetes | Spirochaetia | Spirochaetales | Spirochaetaceae | Sediminispirochaeta | Spirochaeta sp. canine oral taxon 314 |
| 45 | Bacteria | Firmicutes | Clostridia | Clostridiales | Lachnospiraceae | Lachnospiraceae UCG-009 |  |
| 18 | Bacteria | Planctomycetes | Planctomycetacia | Pirellulales | Pirellulaceae | uncultured |  |
| 6889 | Bacteria | Spirochaetes | Spirochaetia | Spirochaetales | Spirochaetaceae | Treponema 2 | Treponema bryantii |
| 2781 | Bacteria | Firmicutes | Erysipelotrichia | Erysipelotrichales | Erysipelotrichaceae | Kandleria | Kandleria vitulina |
| 1293 | Bacteria | Bacteroidetes | Bacteroidia | Bacteroidales | Rikenellaceae | SP3-e08 | uncultured rumen bacterium |
| 300 | Bacteria | Actinobacteria | Coriobacteriia | Coriobacteriales | Coriobacteriales Incertae Sedis | uncultured | uncultured rumen bacterium |
| 23 | Bacteria | Firmicutes | Clostridia | Clostridiales | Ruminococcaceae | uncultured | uncultured Clostridium sp. |
| 17204 | Bacteria | Kiritimatiellaeota | Kiritimatiellae | WCHB1-41 |  |  |  |
| 17 | Bacteria | Verrucomicrobia | Verrucomicrobiae | Verrucomicrobiales | Verrucomicrobiaceae |  |  |
| 29 | Bacteria | Firmicutes | Clostridia | Clostridiales | Clostridiaceae 1 | Clostridium sensu stricto 1 |  |
| 1869 | Bacteria | Firmicutes | Clostridia | Clostridiales | Christensenellaceae | Christensenellaceae R-7 group | uncultured Clostridia bacterium |
| 9 | Bacteria | Firmicutes | Clostridia | Clostridiales | Ruminococcaceae | Caproiciproducens |  |
| 92 | Bacteria | Firmicutes | Clostridia | Clostridiales | Ruminococcaceae | Ruminococcaceae UCG-012 | uncultured rumen bacterium |
| 4 | Bacteria | Patescibacteria | Gracilibacteria | JGI 0000069-P22 |  |  |  |
| 101 | Bacteria | Firmicutes | Clostridia | Clostridiales | Lachnospiraceae | Lachnospiraceae ND3007 group | bacterium V9D2002 |
| 25 | Bacteria | Proteobacteria | Gammaproteobacteria | Pasteurellales | Pasteurellaceae | Haemophilus |  |
| 114 | Bacteria | Bacteroidetes | Bacteroidia | Bacteroidales | M2PB4-65 termite group | uncultured Bacteroidetes bacterium | uncultured Bacteroidetes bacterium |
| 13 | Bacteria | Spirochaetes | Spirochaetia | Spirochaetales | Spirochaetaceae | Treponema 2 | Treponema sp. S |
| 55 | Bacteria | Bacteroidetes | Bacteroidia | Bacteroidales | Porphyromonadaceae | Porphyromonas |  |
| 11081 | Bacteria | Bacteroidetes | Bacteroidia | Bacteroidales | Prevotellaceae | Prevotella 9 | uncultured rumen bacterium |
| 281 | Bacteria | Bacteroidetes | Bacteroidia | Bacteroidales | Prevotellaceae | Prevotellaceae UCG-003 | unidentified rumen bacterium RFN64 |
| 111 | Bacteria | Proteobacteria | Alphaproteobacteria | Acetobacterales | Acetobacteraceae | Acetobacter |  |
| 3686 | Bacteria | Bacteroidetes | Bacteroidia | Bacteroidales | Prevotellaceae | Prevotellaceae UCG-003 | uncultured Bacteroidales bacterium |
| 179 | Bacteria | Proteobacteria | Gammaproteobacteria | Betaproteobacteriales | Burkholderiaceae | Undibacterium |  |
| 215 | Bacteria | Fibrobacteres | Fibrobacteria | Fibrobacterales | Fibrobacteraceae | uncultured | uncultured rumen bacterium |
| 17 | Bacteria | Bacteroidetes | Bacteroidia | Flavobacteriales | Weeksellaceae | Elizabethkingia | Elizabethkingia meningoseptica |
| 35 | Bacteria | Firmicutes | Clostridia | Clostridiales | Lachnospiraceae | FD2005 | uncultured bacterium |
| 658 | Bacteria | Bacteroidetes | Bacteroidia | Bacteroidales | Rikenellaceae |  |  |
| 152 | Bacteria | Actinobacteria | Actinobacteria | Bifidobacteriales | Bifidobacteriaceae | Aeriscardovia | uncultured bacterium |
| 48 | Bacteria | Proteobacteria | Alphaproteobacteria | Rhodospirillales | Rhodopirillaceae | Defluviicoccus |  |
| 232 | Bacteria | Firmicutes | Clostridia | Clostridiales | Ruminococcaceae | Ruminococcaceae UCG-001 | uncultured bacterium |
| 215 | Bacteria | Proteobacteria | Alphaproteobacteria | Azospirillales | Azospirillaceae | Azospirillum |  |
| 52615 | Bacteria | Bacteroidetes | Bacteroidia |  |  |  |  |
| 57 | Bacteria | Cyanobacteria | Melainabacteria | Gastranaerophilales | uncultured rumen bacterium | uncultured rumen bacterium | uncultured rumen bacterium |
| 30 | Bacteria | Planctomycetes | Planctomycetacia | Gemmatales | Gemmataceae | uncultured |  |
| 158 | Bacteria | Firmicutes | Clostridia | Clostridiales | Ruminococcaceae | Ruminococcaceae UCG-014 | unidentified |
| 9105 | Bacteria | Firmicutes | Negativicutes | Selenomonadales | Veillonellaceae | Veillonellaceae UCG-001 | uncultured bacterium |
| 282 | Bacteria | Proteobacteria | Alphaproteobacteria | Rhodospirillales | uncultured | uncultured rumen bacterium | uncultured rumen bacterium |
| 3 | Bacteria | Bacteroidetes | Bacteroidia | Bacteroidales | Prevotellaceae | Prevotella 1 | Prevotella brevis |
| 569 | Bacteria | Firmicutes | Clostridia | Clostridiales | Ruminococcaceae | Ruminococcaceae UCG-002 |  |
| 101 | Bacteria | Firmicutes | Clostridia | Clostridiales | Ruminococcaceae | Ruminococcaceae UCG-013 |  |
| 17 | Bacteria | Bacteroidetes | Bacteroidia | Sphingobacteriales | Lentimicrobiaceae | Lentimicrobium |  |
| 100 | Bacteria | Tenericutes | Mollicutes | Mollicutes RF39 | uncultured Erysipelotrichaceae bacterium | uncultured Erysipelotrichaceae bacterium | uncultured Erysipelotrichaceae bacterium |
| 1964 | Bacteria | Firmicutes | Clostridia | Clostridiales | Ruminococcaceae | Ruminococcaceae UCG-005 |  |
| 3703 | Bacteria | Bacteroidetes | Bacteroidia | Bacteroidales | Bacteroidales RF16 group | uncultured rumen bacterium | uncultured rumen bacterium |
| 9 | Bacteria | Planctomycetes | Planctomycetacia | Pirellulales | Pirellulaceae | Roseimaritima | uncultured bacterium |
| 31 | Bacteria | Spirochaetes | Spirochaetia | Spirochaetales | Spirochaetaceae | M2PT2-76 termite group | uncultured rumen bacterium |
| 5 | Archaea | Euryarchaeota | Thermoplasmata | Methanomassiliicoccales | Methanomethylophilaceae | Candidatus Methanomethylophilus | uncultured archaeon |
| 310 | Bacteria | Firmicutes | Clostridia | Clostridiales | Ruminococcaceae | Ruminiclostridium 5 | uncultured rumen bacterium |
| 82 | Bacteria | Proteobacteria | Alphaproteobacteria | Caulobacterales | Caulobacteraceae | Brevundimonas |  |
| 4 | Bacteria | Proteobacteria | Gammaproteobacteria | Pasteurellales | Pasteurellaceae | Mannheimia | Mannheimia haemolytica |
| 468 | Bacteria | Cyanobacteria | Melainabacteria | Gastranaerophilales | gut metagenome | gut metagenome | gut metagenome |
| 878 | Bacteria | Firmicutes | Clostridia | Clostridiales | Eubacteriaceae | Anaerofustis | uncultured bacterium |
| 418 | Bacteria | Firmicutes | Erysipelotrichia | Erysipelotrichales | Erysipelotrichaceae | Catenisphaera | uncultured rumen bacterium |
| 39 | Bacteria | Firmicutes | Clostridia | Clostridiales | Clostridiales vadinBB60 group | uncultured rumen bacterium 4C28d-15 | uncultured rumen bacterium 4C28d-15 |
| 14 | Bacteria | Firmicutes | Erysipelotrichia | Erysipelotrichales | Erysipelotrichaceae | Erysipelotrichaceae UCG-004 | uncultured Erysipelotrichaceae bacterium |
| 122 | Bacteria | Proteobacteria | Gammaproteobacteria | Aeromonadales | Aeromonadaceae | Aeromonas |  |
| 5658 | Bacteria | Kiritimatiellaeota | Kiritimatiellae | WCHB1-41 | unidentified rumen bacterium RFP12 | unidentified rumen bacterium RFP12 | unidentified rumen bacterium RFP12 |
| 497 | Bacteria | Actinobacteria | Actinobacteria | Corynebacteriales | Corynebacteriaceae | Lawsonella |  |
| 11 | Archaea | Euryarchaeota | Methanobacteria | Methanobacteriales | Methanobacteriaceae | Methanosphaera | uncultured methanogenic archaeon |
| 44 | Bacteria | Fibrobacteres | Fibrobacteria | Fibrobacterales | Fibrobacteraceae | Fibrobacter | Fibrobacter succinogenes subsp. succinogenes |
| 199 | Bacteria | Firmicutes | Bacilli | Bacillales | Bacillaceae | Bacillus |  |
| 28 | Bacteria | Bacteroidetes | Bacteroidia | Bacteroidales | Bacteroidales RF16 group | uncultured Porphyromonadaceae bacterium | uncultured Porphyromonadaceae bacterium |
| 1138 | Bacteria | Firmicutes | Clostridia | Clostridiales | Lachnospiraceae | probable genus 10 | uncultured bacterium |
| 1117 | Bacteria | Proteobacteria | Gammaproteobacteria | Xanthomonadales | Xanthomonadaceae | Stenotrophomonas | Stenotrophomonas rhizophila |
| 39 | Bacteria | Proteobacteria | Alphaproteobacteria | Rhizobiales | Devosiaceae | Pelagibacterium | uncultured alpha proteobacterium |
| 897 | Bacteria | Firmicutes | Clostridia | Clostridiales | Lachnospiraceae | Lachnospiraceae AC2044 group |  |
| 1503 | Bacteria | Firmicutes | Clostridia | Clostridiales | Lachnospiraceae | Lachnospiraceae UCG-002 | uncultured bacterium |
| 11 | Bacteria | Firmicutes | Clostridia | Clostridiales | Lachnospiraceae | uncultured |  |
| 2143 | Bacteria | Firmicutes | Clostridia | Clostridiales | Family XIII | Mogibacterium |  |
| 8 | Bacteria | Firmicutes | Bacilli | Bacillales | Staphylococcaceae | Aliicoccus | uncultured bacterium |
| 568 | Bacteria | Epsilonbacteraeota | Campylobacteria | Campylobacterales | Campylobacteraceae | Campylobacter | uncultured rumen bacterium |
| 25 | Bacteria | Firmicutes | Clostridia |  |  |  |  |
| 72 | Bacteria | Spirochaetes | Spirochaetia | Spirochaetales | Spirochaetaceae | Treponema 2 | uncultured Spirochaeta sp. |
| 253 | Bacteria | Tenericutes | Mollicutes | Mollicutes RF39 | gut metagenome | gut metagenome | gut metagenome |
| 27252 | Bacteria |  |  |  |  |  |  |
| 1034 | Bacteria | Firmicutes | Clostridia | Clostridiales | Ruminococcaceae | Ruminococcaceae NK4A214 group | metagenome |
| 80 | Bacteria | Patescibacteria | Gracilibacteria | Absconditabacteriales (SR1) |  |  |  |
| 186 | Bacteria | Firmicutes | Clostridia | Clostridiales | Ruminococcaceae | Ruminiclostridium | uncultured rumen bacterium |
| 5654 | Bacteria | Cyanobacteria | Oxyphotobacteria | Chloroplast | Ammopiptanthus mongolicus | Ammopiptanthus mongolicus | Ammopiptanthus mongolicus |
| 13 | Bacteria | Planctomycetes | Planctomycetacia | Gemmatales | Gemmataceae | Fimbriiglobus | uncultured Gemmata sp. |
| 26 | Bacteria | Cyanobacteria | Oxyphotobacteria | Oxyphotobacteria Incertae Sedis | Unknown Family | uncultured | uncultured cyanobacterium |
| 8 | Bacteria | Proteobacteria | Gammaproteobacteria | Betaproteobacteriales | Methylophilaceae | Methylotenera |  |
| 3338 | Bacteria | Firmicutes | Clostridia | Clostridiales | Lachnospiraceae | [Eubacterium] ruminantium group |  |
| 29 | Bacteria | Firmicutes | Clostridia | Clostridiales | Ruminococcaceae | UBA1819 |  |
| 577 | Bacteria | Firmicutes | Clostridia | Clostridiales | Ruminococcaceae | Ruminococcaceae UCG-014 | uncultured Ruminococcaceae bacterium |
| 607 | Bacteria | Firmicutes | Clostridia | Clostridiales | Defluviitaleaceae | Defluviitaleaceae UCG-011 | uncultured rumen bacterium |
| 9540 | Archaea | Euryarchaeota | Methanobacteria | Methanobacteriales | Methanobacteriaceae | Methanobrevibacter | uncultured euryarchaeote |
| 11147 | Bacteria | Firmicutes | Clostridia | Clostridiales | Ruminococcaceae | Saccharofermentans | Clostridiales bacterium Firm_14 |
| 58 | Bacteria | Firmicutes | Negativicutes | Selenomonadales | Veillonellaceae | Veillonella |  |
| 156 | Bacteria | Firmicutes | Clostridia | Clostridiales | Ruminococcaceae | Ruminococcus 1 | uncultured Ruminococcus sp. |
| 7 | Bacteria | Bacteroidetes | Bacteroidia | Cytophagales | Hymenobacteraceae | Hymenobacter |  |
| 26 | Bacteria | Chloroflexi | Ktedonobacteria | Ktedonobacterales | Ktedonobacteraceae |  |  |
| 193 | Bacteria | Actinobacteria | Coriobacteriia | Coriobacteriales | Coriobacteriales Incertae Sedis | Raoultibacter |  |
| 9 | Bacteria | Firmicutes | Clostridia | Clostridiales | Lachnospiraceae | Tyzzerella 3 |  |
| 362 | Bacteria | Firmicutes | Clostridia | Clostridiales | Lachnospiraceae | Lachnospiraceae ND3007 | uncultured rumen bacterium |
| 10 | Bacteria | Actinobacteria | Thermoleophilia | Solirubrobacterales | 67-14 |  |  |
| 132 | Bacteria | Firmicutes | Clostridia | Clostridiales | Lachnospiraceae | Lachnospiraceae UCG-002 | uncultured rumen bacterium |
| 639 | Bacteria | Actinobacteria |  |  |  |  |  |
| 29 | Bacteria | Firmicutes | Clostridia | Clostridiales | Lachnospiraceae | Lachnoclostridium 5 |  |
| 184 | Bacteria | Firmicutes | Clostridia | Clostridiales | Lachnospiraceae | Lachnospiraceae NK3A20 group | bacterium ND2018 |
| 9 | Bacteria | Tenericutes | Mollicutes | Mollicutes RF39 | unidentified rumen bacterium RF39 | unidentified rumen bacterium RF39 | unidentified rumen bacterium RF39 |
| 13 | Bacteria | Firmicutes | Clostridia | Clostridiales | Ruminococcaceae | Ethanoligenens | uncultured bacterium |
| 757 | Bacteria | Proteobacteria | Gammaproteobacteria | Betaproteobacteriales | Neisseriaceae | uncultured | uncultured rumen bacterium |
| 43 | Bacteria | Tenericutes | Mollicutes | Mollicutes RF39 | Acholeplasmatales bacterium canine oral taxon 316 | Acholeplasmatales bacterium canine oral taxon 316 | Acholeplasmatales bacterium canine oral taxon 316 |
| 4 | Bacteria | Bacteroidetes | Bacteroidia | Chitinophagales | Chitinophagaceae | Haoranjiania |  |
| 33 | Bacteria | Spirochaetes | Spirochaetia | Spirochaetales | Spirochaetaceae | Treponema 2 | Treponema sp. AC3 |
| 23 | Bacteria | Firmicutes | Clostridia | Clostridiales | Ruminococcaceae | Ruminiclostridium 5 |  |
| 88 | Bacteria | Thermotogae | Thermotogae | Petrotogales | Petrotogaceae | Oceanotoga |  |
| 910 | Bacteria | Actinobacteria | Coriobacteriia | Coriobacteriales |  |  |  |
| 970 | Bacteria | Tenericutes | Mollicutes | Mollicutes RF39 |  |  |  |
| 3 | Bacteria | Firmicutes | Clostridia | Clostridiales | Clostridiaceae 1 |  |  |
| 36 | Bacteria | Proteobacteria | Gammaproteobacteria | Alteromonadales | Shewanellaceae | Shewanella |  |
| 529 | Bacteria | Firmicutes | Clostridia | Clostridiales | Lachnospiraceae | Lachnospiraceae UCG-010 | uncultured rumen bacterium |
| 22 | Bacteria | Synergistetes | Synergistia | Synergistales | Synergistaceae | Pyramidobacter | uncultured bacterium |
| 1240 | Bacteria | Bacteroidetes | Bacteroidia | Bacteroidales | Prevotellaceae | Paraprevotella | uncultured rumen bacterium |
| 175 | Bacteria | Firmicutes | Clostridia | Clostridiales | Lachnospiraceae | Lachnospiraceae NK3A20 group | uncultured Clostridium sp. |
| 742 | Bacteria | Proteobacteria | Gammaproteobacteria | Vibrionales | Vibrionaceae | Salinivibrio |  |
| 52 | Bacteria | Firmicutes | Clostridia | Clostridiales | Peptostreptococcaceae | Romboutsia |  |
| 10 | Bacteria | Actinobacteria | Actinobacteria | Propionibacteriales | Nocardioidaceae | Aeromicrobium |  |
| 2 | Bacteria | Fusobacteria | Fusobacteriia | Fusobacteriales | Leptotrichiaceae | Leptotrichia |  |
| 55 | Bacteria | Bacteroidetes | Bacteroidia | Bacteroidales | Prevotellaceae | Prevotella 1 | unidentified rumen bacterium RFN19 |
| 178 | Bacteria | Proteobacteria | Deltaproteobacteria | Desulfuromonadales | Desulfuromonadaceae |  |  |
| 43 | Bacteria | Proteobacteria | Gammaproteobacteria | Aeromonadales | Succinivibrionaceae | Succinimonas | uncultured rumen bacterium |
| 2557 | Bacteria | Firmicutes | Clostridia | Clostridiales | Lachnospiraceae | [Eubacterium] hallii group | uncultured rumen bacterium |
| 31989 | Bacteria | Actinobacteria | Actinobacteria | Bifidobacteriales | Bifidobacteriaceae |  |  |
| 198 | Bacteria | Proteobacteria | Alphaproteobacteria | Rickettsiales | Mitochondria | uncultured rumen bacterium | uncultured rumen bacterium |
| 442 | Bacteria | Firmicutes | Clostridia | Clostridiales | Lachnospiraceae | Lachnospiraceae UCG-008 | uncultured Lachnospiraceae bacterium |
| 13696 | Bacteria | Firmicutes | Clostridia | Clostridiales | Ruminococcaceae | Ruminococcaceae NK4A214 group |  |
| 408 | Bacteria | Lentisphaerae | Lentisphaeria | Victivallales | Victivallaceae | uncultured rumen bacterium | uncultured rumen bacterium |
| 1006 | Bacteria | Bacteroidetes | Bacteroidia | Bacteroidales | Bacteroidales UCG-001 | uncultured rumen bacterium | uncultured rumen bacterium |
| 42 | Bacteria | Firmicutes | Clostridia | Clostridiales | Ruminococcaceae | Ruminococcaceae UCG-001 |  |
| 6437 | Bacteria | Bacteroidetes | Bacteroidia | Bacteroidales | uncultured | uncultured rumen bacterium | uncultured rumen bacterium |
| 10 | Bacteria | Firmicutes | Clostridia | Clostridiales | Ruminococcaceae | Ruminococcaceae NK4A214 group | unidentified rumen bacterium RF26 |
| 259 | Bacteria | Bacteroidetes | Bacteroidia | Bacteroidales | Prevotellaceae | uncultured bacterium | uncultured bacterium |
| 5359 | Bacteria | Proteobacteria | Gammaproteobacteria | Alteromonadales | Pseudoalteromonadaceae | Pseudoalteromonas |  |
| 38 | Bacteria | Firmicutes | Clostridia | Clostridiales | Lachnospiraceae | Coprococcus 1 |  |
| 209 | Bacteria | Firmicutes | Clostridia | Clostridiales | Lachnospiraceae | Lachnospiraceae UCG-006 | uncultured rumen bacterium |
| 17918 | Bacteria | Firmicutes | Clostridia | Clostridiales |  |  |  |
| 3010 | Bacteria | Bacteroidetes | Bacteroidia | Bacteroidales | Prevotellaceae | Prevotellaceae NK3B31 group | uncultured rumen bacterium |
| 144 | Bacteria | WPS-2 | uncultured rumen bacterium | uncultured rumen bacterium | uncultured rumen bacterium | uncultured rumen bacterium | uncultured rumen bacterium |
| 65 | Bacteria | Chloroflexi | Chloroflexia | Thermomicrobiales | JG30-KF-CM45 |  |  |
| 6 | Bacteria | Chlamydiae | Chlamydiae | Chlamydiales | Parachlamydiaceae |  |  |
| 442 | Bacteria | Bacteroidetes | Bacteroidia | Bacteroidales | Prevotellaceae | Prevotella 1 | Prevotella bryantii |
| 34282 | Bacteria | Bacteroidetes | Bacteroidia | Bacteroidales |  |  |  |
| 88 | Bacteria | Proteobacteria | Gammaproteobacteria | Enterobacteriales | Enterobacteriaceae |  |  |
| 31 | Bacteria | Spirochaetes | Spirochaetia | Spirochaetales | Spirochaetaceae | Sediminispirochaeta |  |
| 41 | Bacteria | Actinobacteria | Actinobacteria | Micrococcales | Micrococcaceae | Kocuria | Kocuria palustris |
| 44 | Bacteria | Bacteroidetes | Bacteroidia | Bacteroidales | Porphyromonadaceae | Porphyromonas | uncultured Porphyromonas sp. |
| 1922 | Bacteria | Lentisphaerae | Oligosphaeria | Oligosphaerales | Oligosphaeraceae | horsej-a03 | uncultured rumen bacterium |
| 8 | Bacteria | Cyanobacteria | Melainabacteria | Gastranaerophilales | uncultured cyanobacterium | uncultured cyanobacterium | uncultured cyanobacterium |
| 14 | Bacteria | Firmicutes | Clostridia | Clostridiales | Lachnospiraceae | Eubacterium uniforme | Eubacterium uniforme |
| 48 | Bacteria | Bacteroidetes | Bacteroidia | Bacteroidales | Bacteroidales RF16 group | unidentified rumen bacterium RFN46 | unidentified rumen bacterium RFN46 |
| 12 | Bacteria | Firmicutes | Clostridia | Clostridiales | Clostridiaceae 1 | Clostridium sensu stricto 1 | Clostridium beijerinckii |
| 561 | Bacteria | Firmicutes | Clostridia | Clostridiales | Christensenellaceae | Christensenellaceae R-7 group | gut metagenome |
| 627 | Bacteria | Bacteroidetes | Bacteroidia | Bacteroidales | Prevotellaceae | Prevotellaceae UCG-003 | unidentified rumen bacterium 12-129 |
| 133 | Bacteria | Firmicutes | Erysipelotrichia | Erysipelotrichales | Erysipelotrichaceae | Asteroleplasma | uncultured Anaeroplasmataceae bacterium |
| 15943 | Bacteria | Firmicutes | Clostridia | Clostridiales | Ruminococcaceae | Ruminococcus 1 |  |
| 18 | Bacteria | Planctomycetes | Planctomycetacia | Pirellulales | Pirellulaceae | Pirellula |  |
| 4 | Bacteria | Verrucomicrobia | Verrucomicrobiae | Pedosphaerales | Pedosphaeraceae | uncultured rumen bacterium | uncultured rumen bacterium |
| 96 | Bacteria | Proteobacteria | Alphaproteobacteria | Paracaedibacterales | Paracaedibacteraceae | uncultured | uncultured rumen bacterium |
| 82 | Bacteria | Bacteroidetes | Bacteroidia | Bacteroidales | Prevotellaceae | uncultured | bacterium AC2042 |
| 646 | Bacteria | Actinobacteria | Coriobacteriia | Coriobacteriales | uncultured | uncultured bacterium | uncultured bacterium |
| 7 | Bacteria | Cyanobacteria | Oxyphotobacteria | Nostocales | Chroococcidiopsaceae | Aliterella CENA595 | uncultured cyanobacterium |
| 51 | Bacteria | Firmicutes | Bacilli | Bacillales | Bacillaceae |  |  |
| 525 | Bacteria | Bacteroidetes | Bacteroidia | Bacteroidales | Prevotellaceae | Alloprevotella | uncultured rumen bacterium |
| 23 | Bacteria | Proteobacteria | Deltaproteobacteria | Desulfuromonadales |  |  |  |
| 22 | Bacteria | Spirochaetes | Spirochaetia | Spirochaetales | Spirochaetaceae | Treponema 2 | Treponema sp. oral taxon 271 |
| 279 | Bacteria | Proteobacteria | Gammaproteobacteria | Betaproteobacteriales | Burkholderiaceae | Comamonas | uncultured rumen bacterium |
| 454 | Bacteria | Synergistetes | Synergistia | Synergistales | Synergistaceae | Fretibacterium | uncultured bacterium |
| 1333 | Bacteria | Actinobacteria | Actinobacteria |  |  |  |  |
| 51 | Bacteria | Proteobacteria | Gammaproteobacteria | Betaproteobacteriales | Burkholderiaceae | Massilia |  |
| 6 | Bacteria | Actinobacteria | Acidimicrobiia | Microtrichales | Iamiaceae | Iamia |  |
| 1308 | Bacteria | Fibrobacteres | Fibrobacteria | Fibrobacterales | Fibrobacteraceae | Fibrobacter | Fibrobacter sp. |
| 113 | Bacteria | Firmicutes | Clostridia | Clostridiales | Family XIII | Family XIII AD3011 group | uncultured rumen bacterium 4C28d-20 |
| 90 | Bacteria | Firmicutes | Clostridia | Clostridiales | Family XIII | Anaerovorax |  |
| 29 | Archaea | Euryarchaeota | Thermoplasmata | Methanomassiliicoccales | Methanomethylophilaceae | uncultured | uncultured Methanobacteriales archaeon |
| 10 | Bacteria | Planctomycetes | Planctomycetacia | Pirellulales | Pirellulaceae | Pirellula | uncultured Pasteuria sp. |
| 105 | Bacteria | Bacteroidetes | Bacteroidia | Sphingobacteriales | Sphingobacteriaceae | Pedobacter |  |
| 68 | Bacteria | Spirochaetes | MVP-15 | Firmicutes bacterium canine oral taxon 309 | Firmicutes bacterium canine oral taxon 309 | Firmicutes bacterium canine oral taxon 309 | Firmicutes bacterium canine oral taxon 309 |
| 54 | Bacteria | Cyanobacteria | Oxyphotobacteria | Chloroplast | Chlorophyta symbiont of Lubomirskia sp. | Chlorophyta symbiont of Lubomirskia sp. | Chlorophyta symbiont of Lubomirskia sp. |
| 24 | Bacteria | Actinobacteria | Actinobacteria | Corynebacteriales | Corynebacteriaceae |  |  |
| 11826 | Bacteria | Proteobacteria | Gammaproteobacteria | Pseudomonadales | Moraxellaceae | Acinetobacter |  |
| 69 | Bacteria | Firmicutes | Clostridia | Clostridiales | Clostridiales vadinBB60 group | uncultured rumen bacterium | uncultured rumen bacterium |
| 189 | Bacteria | Firmicutes | Clostridia | Clostridiales | Lachnospiraceae | Lachnospiraceae NK3A20 group | bacterium AC3007 |
| 1290 | Bacteria | Firmicutes | Clostridia | Clostridiales | Lachnospiraceae | Acetitomaculum | uncultured rumen bacterium 6C3d-13 |
| 723 | Bacteria | Firmicutes | Clostridia | Clostridiales | Lachnospiraceae | Marvinbryantia | uncultured rumen bacterium |
| 404 | Bacteria | Proteobacteria | Deltaproteobacteria | Desulfobacterales | Desulfobulbaceae | Desulfobulbus |  |
| 41 | Bacteria | Bacteroidetes | Bacteroidia | Bacteroidales | Prevotellaceae | Prevotellaceae NK3B31 group | rumen bacterium NK3B31 |
| 51 | Bacteria | Bacteroidetes | Bacteroidia | Bacteroidales | Prevotellaceae | Prevotella 1 | unidentified rumen bacterium 12-130 |
| 7 | Bacteria | Acidobacteria | Acidobacteriia | Acidobacteriales | uncultured |  |  |
| 286 | Bacteria | Proteobacteria | Alphaproteobacteria | Rhodobacterales | Rhodobacteraceae |  |  |
| 4 | Bacteria | Firmicutes | Clostridia | Clostridiales | Ruminococcaceae | Ruminococcaceae UCG-005 | uncultured prokaryote |
| 3015 | Bacteria | Bacteroidetes | Bacteroidia | Bacteroidales | Prevotellaceae | uncultured | uncultured rumen bacterium |
| 2292 | Bacteria | Proteobacteria | Gammaproteobacteria | Enterobacteriales | Enterobacteriaceae | Enterobacter | Lelliottia amnigena |
| 2450 | Bacteria | Proteobacteria | Deltaproteobacteria | Desulfovibrionales | Desulfovibrionaceae | Desulfovibrio | uncultured rumen bacterium |
| 635 | Bacteria | Firmicutes | Clostridia | Clostridiales | Lachnospiraceae | [Eubacterium] hallii group | unidentified rumen bacterium RF24 |
| 3276 | Bacteria | Firmicutes | Clostridia | Clostridiales | Lachnospiraceae | Blautia |  |
| 524 | Bacteria | Firmicutes | Erysipelotrichia | Erysipelotrichales | Erysipelotrichaceae | [Anaerorhabdus] furcosa group | uncultured rumen bacterium |
| 67 | Bacteria | Proteobacteria | Alphaproteobacteria | Sphingomonadales | Sphingomonadaceae |  |  |
| 9 | Bacteria | Actinobacteria | Actinobacteria | Micrococcales | Microbacteriaceae | Leucobacter |  |
| 236 | Bacteria | Bacteroidetes | Bacteroidia | Bacteroidales | Rikenellaceae | Rikenellaceae RC9 gut group | unidentified rumen bacterium RF14 |
| 898 | Bacteria | Firmicutes | Clostridia | Clostridiales | Family XIII | [Eubacterium] nodatum group | Eubacterium sp. AB3007 |
| 1220 | Bacteria | Firmicutes | Clostridia | Clostridiales | Lachnospiraceae | [Eubacterium] cellulosolvens group |  |
| 13 | Bacteria | Proteobacteria | Alphaproteobacteria | Caulobacterales | Caulobacteraceae | Brevundimonas | Brevundimonas olei |
| 4 | Bacteria | Proteobacteria | Alphaproteobacteria | Rhizobiales | Rhizobiaceae | Aminobacter | uncultured bacterium |
| 79 | Bacteria | Proteobacteria | Gammaproteobacteria | Betaproteobacteriales | Burkholderiaceae | Achromobacter |  |
| 4238 | Bacteria | Firmicutes | Clostridia | Clostridiales | Lachnospiraceae | [Eubacterium] ventriosum group | uncultured rumen bacterium |
| 15 | Bacteria | Bacteroidetes | Bacteroidia | Bacteroidales | Porphyromonadaceae | Porphyromonas | unidentified |
| 451 | Bacteria | Firmicutes | Negativicutes | Selenomonadales | Veillonellaceae | Schwartzia |  |
| 927 | Bacteria | Firmicutes | Clostridia | Clostridiales | Ruminococcaceae | Ruminococcaceae UCG-010 |  |
| 57 | Bacteria | Proteobacteria | Gammaproteobacteria |  |  |  |  |
| 2534 | Bacteria | Actinobacteria | Coriobacteriia | Coriobacteriales | Atopobiaceae | Atopobium | uncultured rumen bacterium |
| 834 | Bacteria | Proteobacteria | Gammaproteobacteria | Aeromonadales | Succinivibrionaceae | Ruminobacter | uncultured rumen bacterium |
| 50 | Bacteria | Bacteroidetes | Bacteroidia | Bacteroidales | Prevotellaceae | Prevotellaceae UCG-001 | uncultured rumen bacterium |
| 25 | Bacteria | Chloroflexi | Chloroflexia | Thermomicrobiales | JG30-KF-CM45 | uncultured soil bacterium | uncultured soil bacterium |
| 1775 | Bacteria | Firmicutes | Negativicutes | Selenomonadales | Veillonellaceae | Anaerovibrio | uncultured rumen bacterium |
| 11 | Bacteria | Firmicutes | Clostridia | Clostridiales | Ruminococcaceae | Intestinimonas | uncultured Firmicutes bacterium |
| 8703 | Bacteria | Fibrobacteres | Fibrobacteria | Fibrobacterales | Fibrobacteraceae | Fibrobacter |  |
| 8 | Bacteria | Proteobacteria | Alphaproteobacteria | Rickettsiales | Rickettsiaceae |  |  |
| 328 | Bacteria | Firmicutes | Clostridia | Clostridiales | Lachnospiraceae | [Eubacterium] xylanophilum group | uncultured rumen bacterium |
| 42 | Bacteria | Spirochaetes | MVP-15 | uncultured rumen bacterium | uncultured rumen bacterium | uncultured rumen bacterium | uncultured rumen bacterium |
| 25 | Bacteria | Actinobacteria | Actinobacteria | Micrococcales | Microbacteriaceae | Microbacterium |  |
| 9272 | Bacteria | Firmicutes | Clostridia | Clostridiales | Family XIII | Family XIII AD3011 group | uncultured rumen bacterium |
| 146 | Bacteria | Firmicutes | Clostridia | Clostridiales | Ruminococcaceae | Ruminococcus 1 | rumen bacterium NK4B29 |
| 123 | Bacteria | Proteobacteria | Alphaproteobacteria | Rhizobiales | Beijerinckiaceae | Bosea |  |
| 761 | Bacteria | Firmicutes | Bacilli | Lactobacillales |  |  |  |
| 13 | Bacteria | Actinobacteria | Coriobacteriia | Coriobacteriales | Eggerthellaceae | Denitrobacterium | Denitrobacterium detoxificans |
| 17 | Bacteria | Proteobacteria | Alphaproteobacteria | Rhizobiales | Rhizobiaceae | Mesorhizobium |  |
| 10 | Bacteria | Actinobacteria | Actinobacteria | Actinomycetales | Actinomycetaceae | uncultured |  |
| 481 | Bacteria | Firmicutes | Clostridia | Clostridiales | Ruminococcaceae | Ruminococcus 2 | uncultured organism |
| 9478 | Bacteria | Firmicutes | Clostridia | Clostridiales | Ruminococcaceae | [Eubacterium] coprostanoligenes group |  |
| 755 | Bacteria | Firmicutes | Clostridia | Clostridiales | Ruminococcaceae | Ruminococcus 1 | Ruminococcus flavefaciens |
| 65 | Bacteria | Proteobacteria | Alphaproteobacteria | Rhizobiales | Xanthobacteraceae | Bradyrhizobium |  |
| 13 | Bacteria | Actinobacteria | Actinobacteria | Streptomycetales | Streptomycetaceae |  |  |
| 184 | Bacteria | Firmicutes | Clostridia | Clostridiales | Ruminococcaceae | Ruminiclostridium 9 | uncultured rumen bacterium |
| 481 | Bacteria | Firmicutes | Negativicutes | Selenomonadales | Veillonellaceae | Quinella | uncultured rumen bacterium |
| 4 | Bacteria | Patescibacteria | Saccharimonadia | Saccharimonadales | Saccharimonadaceae | uncultured Candidatus Saccharibacteria bacterium | uncultured Candidatus Saccharibacteria bacterium |
| 5 | Bacteria | Proteobacteria | Alphaproteobacteria | Rhizobiales | Xanthobacteraceae |  |  |
| 760 | Bacteria | Proteobacteria | Alphaproteobacteria | Rhodospirillales | uncultured | gut metagenome | gut metagenome |
| 114 | Bacteria | Kiritimatiellaeota | Kiritimatiellae | WCHB1-41 | uncultured rumen bacterium | uncultured rumen bacterium | uncultured rumen bacterium |
| 5104 | Bacteria | Firmicutes | Clostridia | Clostridiales | Family XIII | Family XIII UCG-001 | uncultured rumen bacterium |
| 32 | Bacteria | Firmicutes | Clostridia | Clostridiales | Lachnospiraceae | Lachnospiraceae FCS020 group |  |
| 11 | Bacteria | Proteobacteria | Gammaproteobacteria | Enterobacteriales | Enterobacteriaceae | Escherichia-Shigella |  |
| 2120 | Bacteria | Firmicutes | Clostridia | Clostridiales | Ruminococcaceae | Ruminococcus 1 | Ruminococcus albus |
| 9804 | Bacteria | Proteobacteria | Gammaproteobacteria | Aeromonadales | Succinivibrionaceae |  |  |
| 131 | Bacteria | Bacteroidetes | Bacteroidia | Flavobacteriales | Flavobacteriaceae | Flavobacterium |  |
| 11 | Bacteria | Proteobacteria | Gammaproteobacteria | Alteromonadales | Idiomarinaceae | Aliidiomarina |  |
| 70 | Bacteria | Firmicutes | Bacilli | Lactobacillales | Streptococcaceae | Streptococcus | Streptococcus salivarius subsp. salivarius |
| 544 | Bacteria | Firmicutes | Clostridia | Clostridiales | Lachnospiraceae | Roseburia |  |
| 4 | Bacteria | Firmicutes | Bacilli |  |  |  |  |
| 43 | Bacteria | Proteobacteria | Gammaproteobacteria | Betaproteobacteriales | Burkholderiaceae | Herbaspirillum |  |
| 80 | Bacteria | Firmicutes | Erysipelotrichia | Erysipelotrichales | Erysipelotrichaceae |  |  |
| 18 | Bacteria | Proteobacteria | Gammaproteobacteria | Diplorickettsiales | Diplorickettsiaceae | Aquicella |  |
| 1171 | Bacteria | Firmicutes | Erysipelotrichia | Erysipelotrichales | Erysipelotrichaceae | Erysipelotrichaceae UCG-009 | uncultured rumen bacterium |
| 15 | Bacteria | Bacteroidetes | Bacteroidia | Bacteroidales | Prevotellaceae | Prevotella 1 | unidentified rumen bacterium RFN93 |
| 8376 | Bacteria | Firmicutes | Clostridia | Clostridiales | Ruminococcaceae |  |  |
| 419 | Bacteria | Firmicutes | Clostridia | Clostridiales | Lachnospiraceae | Lachnospiraceae NK3A20 group | bacterium WCD3001 |
| 521 | Bacteria | Cyanobacteria | Melainabacteria | Gastranaerophilales |  |  |  |
| 457 | Bacteria | Firmicutes | Bacilli | Bacillales | Staphylococcaceae | Staphylococcus |  |
| 182 | Bacteria | Actinobacteria | Actinobacteria | Micrococcales | Micrococcaceae | Micrococcus |  |
| 3798 | Bacteria | Firmicutes | Clostridia | Clostridiales | Ruminococcaceae | Ruminococcaceae UCG-004 | uncultured rumen bacterium |
| 1928 | Bacteria | Firmicutes | Bacilli | Lactobacillales | Lactobacillaceae | Lactobacillus |  |
| 341 | Bacteria | Proteobacteria | Gammaproteobacteria | Enterobacteriales | Enterobacteriaceae | Pectobacterium |  |
| 358 | Bacteria | Proteobacteria | Gammaproteobacteria | Betaproteobacteriales | Burkholderiaceae | Sutterella | gut metagenome |
| 536 | Bacteria | Tenericutes | Mollicutes | Anaeroplasmatales | Anaeroplasmataceae | Anaeroplasma | uncultured rumen bacterium |
| 1249 | Bacteria | Proteobacteria | Alphaproteobacteria | Rhizobiales | Rhizobiaceae | Allorhizobium-Neorhizobium-Pararhizobium-Rhizobium |  |
| 7124 | Bacteria | Firmicutes | Clostridia | Clostridiales | Family XIII |  |  |
| 13 | Bacteria | Proteobacteria | Alphaproteobacteria | Rhizobiales | Rhizobiaceae | Aureimonas |  |
| 558 | Bacteria | Bacteroidetes | Bacteroidia | Bacteroidales | Rikenellaceae | Rikenellaceae RC9 gut group | gut metagenome |
| 813 | Bacteria | Firmicutes | Clostridia | Clostridiales | Lachnospiraceae | Howardella | uncultured rumen bacterium |
| 19 | Bacteria | Patescibacteria | Gracilibacteria | JGI 0000069-P22 | uncultured rumen bacterium | uncultured rumen bacterium | uncultured rumen bacterium |
| 181 | Bacteria | Firmicutes | Clostridia | Clostridiales | Lachnospiraceae | Lachnospiraceae FCS020 group | uncultured rumen bacterium |
| 10 | Bacteria | Firmicutes | Negativicutes | Selenomonadales | Acidaminococcaceae | Succiniclasticum | uncultured rumen bacterium |
| 76 | Archaea | Euryarchaeota | Thermoplasmata | Methanomassiliicoccales | Methanomethylophilaceae | Candidatus Methanomethylophilus | uncultured methanogenic archaeon |
| 14 | Bacteria | Firmicutes | Negativicutes | Selenomonadales | Veillonellaceae | Schwartzia | uncultured bacterium |
| 3992 | Bacteria | Patescibacteria | Saccharimonadia | Saccharimonadales | Saccharimonadaceae | Candidatus Saccharimonas | uncultured Candidatus Saccharibacteria bacterium |
| 12 | Bacteria | Firmicutes | Clostridia | Clostridiales | Clostridiaceae 1 | Clostridium sensu stricto 13 |  |
| 240 | Bacteria | Bacteroidetes | Bacteroidia | Sphingobacteriales | Sphingobacteriaceae |  |  |
| 11 | Bacteria | Proteobacteria | Gammaproteobacteria | Pseudomonadales | Moraxellaceae | Moraxella | Moraxella catarrhalis |
| 518 | Bacteria | Bacteroidetes | Bacteroidia | Bacteroidales | Prevotellaceae | Prevotellaceae UCG-001 | bacterium P3 |
| 190 | Bacteria | Actinobacteria | Actinobacteria | Micrococcales | Intrasporangiaceae |  |  |
| 1242 | Bacteria | Firmicutes | Clostridia | Clostridiales | Lachnospiraceae | [Eubacterium] oxidoreducens group | uncultured rumen bacterium |
| 129 | Bacteria | Actinobacteria | Actinobacteria | Micrococcales | Micrococcaceae |  |  |
| 651 | Bacteria | Lentisphaerae | Oligosphaeria | Oligosphaerales | Oligosphaeraceae | horsej-a03 |  |
| 59 | Bacteria | Proteobacteria | Gammaproteobacteria | Xanthomonadales | Xanthomonadaceae | Luteimonas |  |
| 744 | Bacteria | Firmicutes | Clostridia | Clostridiales | Lachnospiraceae | [Eubacterium] ruminantium group | uncultured Lachnospiraceae bacterium |
| 67 | Bacteria | Firmicutes | Clostridia | Clostridiales | Lachnospiraceae | Tyzzerella 3 | uncultured rumen bacterium |
| 103 | Bacteria | Proteobacteria | Gammaproteobacteria | Aeromonadales | Succinivibrionaceae | Succinimonas |  |
| 7 | Bacteria | Proteobacteria | Gammaproteobacteria | Betaproteobacteriales | Burkholderiaceae | Cupriavidus |  |
| 125 | Bacteria | Firmicutes | Clostridia | Clostridiales | Family XIII | [Eubacterium] nodatum group | uncultured rumen bacterium |
| 3083 | Bacteria | Spirochaetes | Spirochaetia | Spirochaetales | Spirochaetaceae | Sediminispirochaeta | uncultured rumen bacterium |
| 282 | Bacteria | Firmicutes | Negativicutes | Selenomonadales | Veillonellaceae | Quinella | uncultured bacterium |
| 119 | Bacteria | Firmicutes | Bacilli | Bacillales | Bacillaceae | Anoxybacillus |  |
| 3936 | Bacteria | Firmicutes | Bacilli | Lactobacillales | Streptococcaceae | Streptococcus |  |
| 104 | Bacteria | Firmicutes | Clostridia | Clostridiales | Peptostreptococcaceae | Paeniclostridium |  |
| 6 | Bacteria | Patescibacteria | ABY1 | Candidatus Falkowbacteria | uncultured Parcubacteria group bacterium | uncultured Parcubacteria group bacterium | uncultured Parcubacteria group bacterium |
| 20060 | Bacteria | Actinobacteria | Actinobacteria | Bifidobacteriales | Bifidobacteriaceae | Bifidobacterium |  |
| 767 | Bacteria | Firmicutes | Clostridia | Clostridiales | Lachnospiraceae | Oribacterium |  |
| 6 | Bacteria | Firmicutes | Clostridia | Clostridiales | Lachnospiraceae | Agathobacter | uncultured rumen bacterium |
| 23 | Bacteria | Chloroflexi | Ktedonobacteria | Ktedonobacterales | Ktedonobacteraceae | HSB OF53-F07 |  |
| 22 | Bacteria | Firmicutes | Clostridia | Clostridiales | Lachnospiraceae | Coprococcus 2 | uncultured rumen bacterium |
| 84 | Bacteria | Bacteroidetes | Bacteroidia | Flavobacteriales | Weeksellaceae | Elizabethkingia |  |
| 505 | Bacteria | Bacteroidetes | Bacteroidia | Bacteroidales | Bacteroidaceae | Bacteroides |  |
| 942 | Bacteria | Firmicutes | Clostridia | Clostridiales | Lachnospiraceae | Lachnoclostridium 1 | uncultured rumen bacterium |
| 633 | Bacteria | Spirochaetes | Spirochaetia | Spirochaetales | Spirochaetaceae | Treponema 2 | bacterium WCE3006 |
| 762 | Bacteria | Proteobacteria | Alphaproteobacteria | Rhizobiales | Rhizobiaceae | Ochrobactrum |  |
| 58 | Bacteria | Firmicutes | Clostridia | Clostridiales | Ruminococcaceae | Ruminococcaceae UCG-010 | uncultured organism |
| 825 | Bacteria | Proteobacteria |  |  |  |  |  |
| 30 | Bacteria | Actinobacteria | Thermoleophilia | Solirubrobacterales | Solirubrobacteraceae | uncultured |  |
| 28 | Bacteria | Proteobacteria | Gammaproteobacteria | Betaproteobacteriales | Methylophilaceae | Methylophilus | uncultured Methylophilus sp. |
| 735 | Bacteria | Bacteroidetes | Bacteroidia | Bacteroidales | Prevotellaceae | Prevotellaceae UCG-004 | uncultured rumen bacterium |
| 19 | Bacteria | Patescibacteria | Saccharimonadia | Saccharimonadales | uncultured gamma proteobacterium | uncultured gamma proteobacterium | uncultured gamma proteobacterium |
| 2884 | Bacteria | Lentisphaerae | Lentisphaeria | Victivallales | vadinBE97 | uncultured bacterium | uncultured bacterium |
| 15 | Bacteria | Planctomycetes | Planctomycetacia | Planctomycetales | uncultured | uncultured Planctomyces sp. | uncultured Planctomyces sp. |
| 1508 | Bacteria | Actinobacteria | Coriobacteriia | Coriobacteriales | Eggerthellaceae |  |  |
| 28 | Bacteria | Bacteroidetes | Bacteroidia | Flavobacteriales | Weeksellaceae |  |  |
| 16 | Bacteria | Deinococcus-Thermus | Deinococci | Deinococcales | Deinococcaceae | Deinococcus |  |
| 3869 | Bacteria | Proteobacteria | Gammaproteobacteria | Pseudomonadales | Pseudomonadaceae | Pseudomonas |  |
| 20 | Bacteria | Firmicutes | Erysipelotrichia | Erysipelotrichales | Erysipelotrichaceae | Turicibacter | uncultured organism |
| 8 | Bacteria | Patescibacteria | WS6 (Dojkabacteria) | uncultured anaerobic bacterium | uncultured anaerobic bacterium | uncultured anaerobic bacterium | uncultured anaerobic bacterium |
| 1785 | Bacteria | Proteobacteria | Gammaproteobacteria | Pseudomonadales | Moraxellaceae | Acinetobacter | Acinetobacter indicus |
| 11424 | Bacteria | Bacteroidetes | Bacteroidia | Bacteroidales | Prevotellaceae | Prevotella 1 | Prevotella ruminicola |
| 220 | Bacteria | Proteobacteria | Alphaproteobacteria | Rhizobiales | Beijerinckiaceae | Methylobacterium | uncultured alpha proteobacterium |
| 10 | Bacteria | Firmicutes | Clostridia | Clostridiales | Ruminococcaceae | Ruminococcaceae V9D2013 group |  |
| 19 | Bacteria | Actinobacteria | Actinobacteria | Micrococcales | Micrococcaceae | Glutamicibacter |  |
| 6160 | Bacteria | Firmicutes | Clostridia | Clostridiales | Ruminococcaceae | Ruminococcus 2 |  |
| 2209 | Archaea | Euryarchaeota | Methanobacteria | Methanobacteriales | Methanobacteriaceae | Methanobrevibacter |  |
| 59 | Bacteria | Actinobacteria | Actinobacteria | Frankiales | Geodermatophilaceae | Geodermatophilus |  |
| 79 | Bacteria | Actinobacteria | Coriobacteriia | Coriobacteriales | Atopobiaceae | uncultured | uncultured rumen bacterium |
| 126 | Bacteria | Firmicutes | Bacilli | Lactobacillales | Lactobacillaceae | Lactobacillus | uncultured Firmicutes bacterium |
| 1155 | Bacteria | Bacteroidetes | Bacteroidia | Bacteroidales | Bacteroidales RF16 group |  |  |
| 19 | Bacteria | Planctomycetes | Planctomycetacia | Isosphaerales | Isosphaeraceae | Singulisphaera | uncultured Planctomycetaceae bacterium |
| 60 | Bacteria | Firmicutes | Clostridia | Clostridiales | Lachnospiraceae | Lachnospiraceae NK3A20 group | uncultured Lachnospiraceae bacterium |
| 13 | Bacteria | Fusobacteria | Fusobacteriia | Fusobacteriales | Leptotrichiaceae | Leptotrichia | uncultured Leptotrichia sp. |
| 306 | Bacteria | Firmicutes | Negativicutes | Selenomonadales | Veillonellaceae | Quinella |  |
| 15 | Bacteria | Firmicutes | Clostridia | Clostridiales | Lachnospiraceae | Lachnospiraceae NK3A20 group | uncultured Firmicutes bacterium |
| 898 | Bacteria | Proteobacteria | Gammaproteobacteria | Enterobacteriales | Enterobacteriaceae | Serratia | Serratia nematodiphila |
| 456 | Bacteria | Proteobacteria | Alphaproteobacteria | Rhodospirillales | uncultured |  |  |
| 102 | Bacteria | Proteobacteria | Alphaproteobacteria | Rhizobiales | Beijerinckiaceae | Methylobacterium |  |
| 1384 | Bacteria | Firmicutes | Clostridia | Clostridiales | Lachnospiraceae | Pseudobutyrivibrio |  |
| 51 | Bacteria | Proteobacteria | Gammaproteobacteria | Betaproteobacteriales | Burkholderiaceae | Tepidimonas |  |
| 77 | Bacteria | Proteobacteria | Alphaproteobacteria | Acetobacterales | Acetobacteraceae | Roseomonas |  |
| 44 | Bacteria | Firmicutes | Clostridia | Clostridiales | Ruminococcaceae | Sporobacter |  |
| 36 | Bacteria | Proteobacteria | Alphaproteobacteria | Rickettsiales | Anaplasmataceae | Anaplasma | Anaplasma marginale |
| 224 | Bacteria | Firmicutes | Clostridia | Clostridiales | Lachnospiraceae | Lachnospiraceae UCG-008 | uncultured rumen bacterium |
| 577 | Bacteria | Bacteroidetes | Bacteroidia | Bacteroidales | Muribaculaceae |  |  |
| 198 | Bacteria | Proteobacteria | Alphaproteobacteria | Rickettsiales | Mitochondria |  |  |
| 569 | Bacteria | Lentisphaerae | Lentisphaeria | Victivallales | vadinBE97 | uncultured rumen bacterium | uncultured rumen bacterium |
| 3806 | Bacteria | Firmicutes | Clostridia | Clostridiales | Lachnospiraceae | [Eubacterium] hallii group |  |
| 19 | Bacteria | Proteobacteria | Gammaproteobacteria | Pasteurellales | Pasteurellaceae |  |  |
| 159 | Bacteria | Firmicutes | Clostridia | Clostridiales | Ruminococcaceae | Ruminococcaceae V9D2013 group | uncultured rumen bacterium |
| 48 | Bacteria | Tenericutes | Mollicutes | NED5E9 | uncultured rumen bacterium | uncultured rumen bacterium | uncultured rumen bacterium |
| 43 | Bacteria | Proteobacteria | Gammaproteobacteria | Cardiobacteriales | Cardiobacteriaceae | Suttonella |  |
| 7 | Bacteria | Bacteroidetes | Bacteroidia | Bacteroidales | Prevotellaceae | Prevotella 2 | unidentified |
| 38 | Bacteria | Firmicutes | Clostridia | Clostridiales | Lachnospiraceae | Lachnospiraceae NK4A136 group | bacterium XPB1013 |
| 7 | Bacteria | Firmicutes | Clostridia | Clostridiales | Lachnospiraceae | Lachnospiraceae NK3A20 group | Lachnospiraceae bacterium KH1T2 |
| 5934 | Bacteria | Planctomycetes | Planctomycetacia | Pirellulales | Pirellulaceae | CPla-4 termite group | uncultured rumen bacterium |
| 8 | Bacteria | Bacteroidetes | Bacteroidia | Bacteroidales | Tannerellaceae | Parabacteroides |  |
| 5242 | Bacteria | Firmicutes | Clostridia | Clostridiales | Ruminococcaceae | Ruminococcaceae UCG-014 |  |
| 556 | Bacteria | Bacteroidetes | Bacteroidia | Bacteroidales | Prevotellaceae | Prevotella 1 | unidentified rumen bacterium RC17 |
| 32 | Bacteria | Synergistetes | Synergistia | Synergistales | Synergistaceae |  |  |
| 50 | Bacteria | Bacteroidetes | Bacteroidia | Bacteroidales | Rikenellaceae | hoa5-07d05 gut group | uncultured Bacteroidales bacterium |
| 29316 | Bacteria | Bacteroidetes | Bacteroidia | Bacteroidales | Rikenellaceae | Rikenellaceae RC9 gut group |  |
| 5096 | Bacteria | Firmicutes | Clostridia | Clostridiales | Ruminococcaceae | Saccharofermentans | uncultured microorganism |
| 6 | Bacteria | Firmicutes | Clostridia | Clostridiales | Family XIII | Mogibacterium | uncultured rumen bacterium 3C0d-4 |
| 68 | Bacteria | Planctomycetes | Planctomycetacia | Planctomycetales | Gimesiaceae | uncultured | uncultured Planctomyces sp. |
| 3 | Bacteria | Bacteroidetes | Bacteroidia | Bacteroidales | Dysgonomonadaceae | Dysgonomonas |  |
| 16 | Bacteria | Lentisphaerae | Oligosphaeria | Oligosphaerales | Oligosphaeraceae |  |  |
| 1538 | Bacteria | Bacteroidetes | Bacteroidia | Bacteroidales | Prevotellaceae | Prevotellaceae Ga6A1 group | uncultured rumen bacterium |
| 37 | Bacteria | Bacteroidetes | Bacteroidia | Bacteroidales | Prevotellaceae | Prevotella 1 | unidentified rumen bacterium RFN92 |
| 2314 | Bacteria | Proteobacteria | Gammaproteobacteria | Betaproteobacteriales | Neisseriaceae |  |  |
| 19 | Bacteria | Tenericutes | Mollicutes | Mollicutes RF39 | metagenome | metagenome | metagenome |
| 138 | Archaea | Euryarchaeota | Thermoplasmata | Methanomassiliicoccales | Methanomethylophilaceae | uncultured |  |
| 3595 | Bacteria | Firmicutes | Clostridia | Clostridiales | Lachnospiraceae | Oribacterium | uncultured rumen bacterium |
| 278 | Bacteria | Firmicutes | Clostridia | Clostridiales | Lachnospiraceae | Moryella | bacterium YAD2002 |
| 2358 | Bacteria | Firmicutes | Clostridia | Clostridiales | Lachnospiraceae | Moryella | uncultured rumen bacterium |
| 1560 | Bacteria | Firmicutes | Clostridia | Clostridiales | Lachnospiraceae | Lachnospiraceae XPB1014 group |  |
| 2891 | Bacteria | Synergistetes | Synergistia | Synergistales | Synergistaceae | Pyramidobacter | uncultured rumen bacterium |
| 5 | Bacteria | Proteobacteria | Gammaproteobacteria | Aeromonadales | Succinivibrionaceae | uncultured |  |
| 41 | Bacteria | Bacteroidetes | Bacteroidia | Bacteroidales | Rikenellaceae | Blvii28 wastewater-sludge group |  |
| 10 | Bacteria | Bacteroidetes | Bacteroidia | Bacteroidales | Paludibacteraceae | uncultured | uncultured rumen bacterium |
| 225 | Bacteria | Firmicutes | Clostridia | Clostridiales | Ruminococcaceae | Ruminococcaceae UCG-010 | gut metagenome |
| 87 | Bacteria | Bacteroidetes | Bacteroidia | Flavobacteriales | Weeksellaceae | Empedobacter | uncultured bacterium |
| 8 | Bacteria | Proteobacteria | Alphaproteobacteria | Sphingomonadales | Sphingomonadaceae | Rhizorhapis |  |
| 11 | Bacteria | Firmicutes | Clostridia | Clostridiales | Lachnospiraceae | Shuttleworthia | uncultured rumen bacterium |
| 9691 | Bacteria | Patescibacteria | Saccharimonadia | Saccharimonadales | Saccharimonadaceae | Candidatus Saccharimonas |  |
| 910 | Bacteria | Cyanobacteria | Oxyphotobacteria | Chloroplast |  |  |  |
| 209 | Bacteria | Bacteroidetes | Bacteroidia | Bacteroidales | Prevotellaceae | Prevotellaceae UCG-001 | uncultured Prevotella sp. |
| 13 | Bacteria | Firmicutes | Clostridia | Clostridiales | Clostridiales vadinBB60 group |  |  |
| 42 | Bacteria | Bacteroidetes | Bacteroidia | Bacteroidales | Dysgonomonadaceae |  |  |
| 16805 | Bacteria | Bacteroidetes | Bacteroidia | Bacteroidales | Prevotellaceae | Prevotella 1 |  |
| 2265 | Bacteria | Elusimicrobia | Elusimicrobia | Elusimicrobiales | Elusimicrobiaceae | Elusimicrobium | uncultured bacterium |
| 59 | Bacteria | Firmicutes | Clostridia | Clostridiales | Peptococcaceae | uncultured | uncultured rumen bacterium |
| 13885 | Bacteria | Firmicutes | Clostridia | Clostridiales | Christensenellaceae | Christensenellaceae R-7 group |  |
| 887 | Bacteria | Proteobacteria | Gammaproteobacteria | Aeromonadales | Succinivibrionaceae | Succinivibrionaceae UCG-002 | uncultured rumen bacterium |
| 117160 | Bacteria | Proteobacteria | Gammaproteobacteria | Enterobacteriales | Enterobacteriaceae | Escherichia-Shigella | unidentified |
| 132 | Bacteria | Actinobacteria | Coriobacteriia | Coriobacteriales | Atopobiaceae | Olsenella | uncultured Olsenella sp. |
| 13 | Bacteria | Firmicutes | Negativicutes | Selenomonadales | Veillonellaceae | Anaerovibrio |  |
| 11 | Bacteria | Proteobacteria | Gammaproteobacteria | Betaproteobacteriales | Burkholderiaceae | Sutterella | uncultured rumen bacterium |
| 24 | Bacteria | Proteobacteria | Gammaproteobacteria | Oceanospirillales | Nitrincolaceae | Nitrincola | uncultured bacterium |
| 30 | Bacteria | Actinobacteria | Actinobacteria | Propionibacteriales | Propionibacteriaceae |  |  |
| 636 | Bacteria | Bacteroidetes | Bacteroidia | Bacteroidales | Prevotellaceae | Prevotellaceae UCG-003 |  |
| 28 | Bacteria | Actinobacteria | Actinobacteria | Propionibacteriales | Propionibacteriaceae | Propioniciclava | metagenome |
| 1307 | Bacteria | Bacteroidetes | Bacteroidia | Bacteroidales | Prevotellaceae | Prevotellaceae UCG-001 |  |
| 30 | Bacteria | Firmicutes | Clostridia | Clostridiales | Lachnospiraceae | [Eubacterium] ruminantium group | bacterium YSB2008 |
| 2 | Bacteria | Actinobacteria | Coriobacteriia | Coriobacteriales | Eggerthellaceae | DNF00809 |  |
| 21 | Bacteria | Firmicutes | Clostridia | Clostridiales | Ruminococcaceae | Oscillospira |  |
| 1475 | Bacteria | Bacteroidetes | Bacteroidia | Bacteroidales | Rikenellaceae | U29-B03 |  |
| 11 | Bacteria | Firmicutes | Clostridia | Clostridiales | Ruminococcaceae | Ruminococcaceae UCG-005 | uncultured organism |
| 12 | Bacteria | Actinobacteria | Actinobacteria | Propionibacteriales | Nocardioidaceae |  |  |
| 490 | Bacteria | Proteobacteria | Gammaproteobacteria | Betaproteobacteriale |  |  |  |
| 190 | Bacteria | Chloroflexi | Anaerolineae | Anaerolineales | Anaerolineaceae | Flexilinea |  |
| 855 | Bacteria | Firmicutes | Clostridia | Clostridiales | Family XIII | [Eubacterium] saphenum group | uncultured bacterium |
| 87 | Bacteria | Firmicutes | Negativicutes | Selenomonadales | Acidaminococcaceae | Succiniclasticum |  |
| 113 | Bacteria | Firmicutes | Bacilli | Bacillales | Planococcaceae | Lysinibacillus |  |
| 424 | Bacteria | Proteobacteria | Gammaproteobacteria | Betaproteobacteriale | Neisseriaceae | uncultured |  |
| 28 | Bacteria | Bacteroidetes | Bacteroidia | Bacteroidales | Prevotellaceae | Prevotellaceae NK3B31group |  |
| 370 | Bacteria | Firmicutes | Clostridia | Clostridiales | Ruminococcaceae | Ruminococcus 1 | uncultured Ruminococcaceae bacterium |
| 11 | Bacteria | Firmicutes | Clostridia | Clostridiales | Christensenellaceae | Christensenellaceae R-7 group | uncultured rumen bacterium 4C0d-14 |
| 1360 | Bacteria | Firmicutes | Clostridia | Clostridiales | Christensenellaceae | Christensenellaceae R-7 group | uncultured rumen bacterium 4C0d-15 |
| 24 | Archaea | Euryarchaeota | Thermoplasmata | Methanomassiliicoccales | Methanomethylophilaceae | uncultured | uncultured rumen methanogen |
| 346 | Bacteria | Proteobacteria | Gammaproteobacteria | Betaproteobacterials | Burkholderiaceae | Delftia |  |
| 259 | Bacteria | Synergistetes | Synergistia | Synergistales | Synergistaceae | Fretibacterium |  |
| 24 | Bacteria | Proteobacteria | Alphaproteobacteria | Rhizobiales | Devosiaceae | Devosia |  |
| 17712 | Bacteria | Proteobacteria | Alphaproteobacteria |  |  |  |  |
| 27 | Bacteria | Firmicutes | Clostridia | Clostridiales | Lachnospiraceae | Lachnobacterium | Lachnobacterium bovis |
| 383 | Bacteria | Firmicutes | Clostridia | Clostridiales | Ruminococcaceae | Ruminiclostridium 6 | uncultured rumen bacterium |
| 23 | Bacteria | Actinobacteria | Actinobacteria | Micrococcales | Intrasporangiaceae | Ornithinimicrobium | uncultured bacterium |
| 65 | Bacteria | Bacteroidetes | Bacteroidia | Bacteroidales | Prevotellaceae | Prevotella 7 | Prevotella melaninogenica |
| 3292 | Bacteria | Firmicutes | Negativicutes | Selenomonadales | Veillonellaceae |  |  |
| 9 | Bacteria | Bacteroidetes | Bacteroidia | Flavobacteriales | Weeksellaceae | Cloacibacterium | uncultured Cloacibacterium sp. |
| 78 | Bacteria | Actinobacteria | Actinobacteria | Corynebacteriales | Mycobacteriaceae | Mycobacterium |  |
| 696 | Bacteria | Firmicutes | Negativicutes | Selenomonadales | Veillonellaceae | Selenomonas |  |
| 5 | Bacteria | Lentisphaerae | Oligosphaeria | Oligosphaerales | Oligosphaeraceae | Z20 | uncultured Lentisphaerae |
| 765 | Bacteria | Firmicutes | Clostridia | Clostridiales | Lachnospiraceae | Lachnoclostridium 10 | uncultured rumen bacterium |
| 65 | Bacteria | Firmicutes | Clostridia | Clostridiales | Lachnospiraceae | XBB1006 | uncultured rumen bacterium |
| 12 | Bacteria | Actinobacteria | Actinobacteria | Propionibacteriales | Propionibacteriaceae | Acidipropionibacterium |  |
| 6878 | Bacteria | Actinobacteria | Coriobacteriia | Coriobacteriales | Atopobiaceae | Olsenella | uncultured rumen bacterium |
| 35870 | Bacteria | Proteobacteria | Gammaproteobacteria | Oceanospirillales | Halomonadaceae | Halomonas |  |
| 8 | Bacteria | Bacteroidetes | Bacteroidia | Bacteroidales | Rikenellaceae | Rikenellaceae RC9 gut group | Eubacterium sp. F1 |
